# Advanced organoid models for targeting *Kras*-driven lung adenocarcinoma in drug discovery and combination therapy

**DOI:** 10.1186/s13046-025-03385-9

**Published:** 2025-04-24

**Authors:** İsa Taş, Ruben Jacobs, Juliane Albrecht, Sebastian A. Barrientos, Josephine Åberg, Wondossen Sime, Hans Brunnström, Helena Persson, Julhash U. Kazi, Ramin Massoumi

**Affiliations:** 1 Department of Laboratory Medicine, Division of Translational Cancer Research, Lund University, Lund, Sweden; 2 Department of Clinical Sciences Lund, Division of Oncology, Lund University, Lund, Sweden; 3 Department of Clinical Sciences Lund, Division of Pathology, Lund University, Lund, Sweden; 4 IVRS AB, Medicon Village, Scheeletorget 1, 223-81 Lund, SE Sweden

**Keywords:** Lung adenocarcinoma, Organoids, *Kras G12V*, Midostaurin, Decitabine

## Abstract

**Background:**

Lung cancer remains one of the most challenging diseases to treat due to its heterogeneity. Kirsten Rat Sarcoma Viral Oncogene Homolog (KRAS) mutations are genetic drivers in numerous cancer types including lung adenocarcinoma (LUAD). Despite recent advances in KRAS-targeted therapies, treatment resistance and limited therapeutic options necessitate advanced preclinical models, such as organoids, to identify personalized cancer therapies by screening novel therapeutic strategies and synergistic drug combinations.

**Results:**

We established LUAD in genetically engineered mouse (GEM) models of *Kras*^G12V^ & *Trp53* ^Δex2−10^ (KP) and KP with *Ctnnb1*^Δex3^ mutation (KPC). Tumor-derived organoids from these models recapitulated the genomic landscape and histopathological characteristics of their parental tumors. The organoids displayed tumorigenic potential when implanted in immunocompromised mice, forming tumors in contrast to unlike healthy lung-derived organoids. Drug screening identified effective kinase inhibitors and DNA methyltransferase (DNMT) inhibitors against the organoids. Notably, the combination of these drugs exhibited the highest synergy in KPC organoids.

**Conclusion:**

We successfully developed LUAD organoids harboring *Kras* mutations and identified multiple potential therapeutic agents targeting these cells. Furthermore, we demonstrated the effectiveness of a DNMT inhibitor-based combination therapy, presenting a promising strategy for this challenging lung cancer subtype.

**Supplementary Information:**

The online version contains supplementary material available at 10.1186/s13046-025-03385-9.

## Introduction

Lung cancer is the leading cause of cancer-related mortality worldwide [1]. Non-small cell lung cancer (NSCLC), accounting for approximately 85% of lung cancer cases, includes squamous cell carcinoma (SCC) and lung adenocarcinoma (LUAD) as the most common subtypes, accounting for over 40% of all lung tumors [2]. Over the past few decades, substantial progress has been made in understanding the biology and progression mechanisms of lung cancer. With the advent of targeted therapies and immunotherapies, promising clinical outcomes have been achieved. However, despite the advancements in diagnosis and treatment, the prognosis for lung cancer patients remains unsatisfactory [2, 3].

KRAS is the most frequently mutated isoform of the RAS family, with approximately 32% of all LUADs being driven by *KRAS* mutations. The most common types of mutations involve amino-acid substitutions including G12C (46%), G12V (23%), and G12D (17%) [4]. Mutant-specific KRAS inhibitors (e.g., KRAS G12C and KRAS G12D inhibitors) and pan-KRAS inhibitors are currently undergoing clinical evaluation and have shown promising therapeutic potential. To date, the U.S. Food and Drug Administration (FDA) has approved only sotorasib (Lumakras™) and adagrasib (Krazati®) for patients harboring the *KRAS G12C* mutation [5, 6]. However, despite the initial efficacy of these first-generation KRAS G12C inhibitors, the emergence of drug resistance and disease progression remains a significant challenge in most patients. Additionally, while pan-KRAS inhibitors aim to target multiple KRAS isoforms, their clinical application is hindered by dose-limiting toxicities associated with wild-type KRAS inhibition. Recently, extensive research efforts are focused on developing second-generation KRAS inhibitors to overcome resistance mechanisms and exploring combination therapy strategies to enhance treatment efficacy and patient outcomes [7, 8]. Unlike *KRAS G12C*, the valine substitution in *KRAS G12V* does not allow for covalent inhibition or the formation of strong polar, non-covalent interactions, making it particularly challenging to target. To address this, the tri-complex inhibitor platform has been developed, which leverages chemical remodeling of the cellular chaperone cyclophilin A (CypA) to engage previously "undruggable" surfaces and enable mutant-selective inhibition. Recently, RM-048, a tri-complex inhibitor specifically targeting the KRAS G12V(ON) state, has entered preclinical investigation [9]. Insufficient therapeutic solutions for KRAS G12V, the second most prevalent *KRAS* mutation in LUAD, underscores the urgent need for novel therapeutic strategies to effectively target this oncogenic driver.

Pre-clinical models are invaluable tools for investigating tumor progression and evaluating the efficacy of therapeutic agents [10]. GEM models have significantly advanced our understanding of tumor initiation, development and metastases [11]. In this study, the GEM model allowed us to generate *Kras* ^G12V^ -driven LUAD, incorporating various clinically relevant co-occurring mutations. Three-dimensional (3D) tumor organoid models are particularly advantageous as they preserve the complex histological architecture, biomarker expression, and mutational spectrum of their parental tumor tissue, making them ideal tools for drug screening. Organoid models have been successfully established for several cancers, including colon, prostate, breast, liver, bladder, and liver cancers. In relation to lung cancer, several studies have developed patient-derived NSCLC organoid models to evaluate drug response [1, 12–15]. However, these studies primarily focus on random sampling, organoid generation, and biobanking, without specifically targeting key molecular drivers of lung cancer. This may be due to limited access to tumor samples with major oncogene mutations and the challenges in successfully developing mutated lung cancer PDOs. Therefore, improving preclinical models, such as organoids, is crucial for more effectively targeting oncogenic drivers like *KRAS* and its subtypes in cancer research and therapy development.

In this study, we have generated GEM models harboring *Kras/Trp53* (KP) and *Kras/Trp53/Ctnnb1* (KPC) mutations which derive the development of murine LUAD. We successfully established and cultured organoids in vitro, followed by comprehensive genetic and histopathological characterization. The tumorigenic potential of the organoids was further validated by in vivo models. The organoids exhibited selective sensitivity to amuvatinib, midostaurin and selumetinib. Additionally, the epigenetic drug, decitabine, showed remarkable synergic activity when combined with midostaurin. The establishment of this innovative organoid platform provides a valuable preclinical model for studying LUAD and offers significant potential for future drug screening endeavors and the identification of therapeutic targets.

## Materials and methods

### Genetically engineered Kras-driven LUAD model

Mice with tamoxifen-inducible Cre recombinase (CreERT2) in the secretoglobin Family 1A Member 1 (*Scgb1a1*) gene locus coding for Clara cells secreted 10KDa protein (CC10) gene locus were obtained from The Jackson Laboratory. The CC10-CreERT2; *Kras*^LSLG12Vgeo/WT^; *Trp*53^F2−10^ (KP) and *Ctnnb1* ^loxEx3^ (KPC) LUAD models were homozygous for the CC10-CreERT2 (B6N.129S6(Cg)*Scgb1a* 1tm1(Cre/ERT)Blh/J) knock-in allele, homozygous for the *Trp53* ^F2−10^ (*Trp53*tm1Brn) knock-in allele, heterozygous for the Kras^LSLG12Vgeo^ knock-in allele, and homozyous for the *Ctnnb1*^loxEx3^ (Ctnnb1 tm1Mmt).

KP model was kindly provided by Dr. Ernesto Bockamp, Institute of Translational Immunology (TIM), University Medical Center, Johannes Gutenberg University, Mainz, Germany. Ctnnb1 (C) model was kindly provided by Dr. MD. Makoto M Taketo, Institute for Advancement of Clinical and Translational Science, Kyoto University Hospital, Japan.

To induce tumor formation, KP and KPC mice received an intra-peritoneal injection of tamoxifen (TAM) (0.2 mg/g per mouse) for 4 consecutive days. All mice were kept up to 6 months following tumor induction before termination unless the humane endpoint was reached before this time-point. All experiments were approved by the Swedish regional (Malmö-Lund) ethical Committee (12,303–23) and performed according to the national and international guidelines of the European Union.

### Genotyping

For genotyping the *Scgb1a1*-CreERT2 allele, we used oligonucleotides 5’-ACTCACTATTGGGGGTGTGG-3’, 5’-AGGCTCCTGGCTGGAATAGT-3’ and 5’-CCAAAAGACGGCAATATGGT-3’ yielding a 245 bp for the mutant and a 550 bp for the wild-type locus. The *Kras* LSLG12Vgeo allele was identified by the oligonucleotides 5’-CGTCCAGCGTGTCCTAGACTTTA-3’, 5’-TGACCGCTTCCTCGTGCTT-3’ and 5’-ACTATTTCATACTGGGTCTGCCTT-3’ yielding a 390 bp for the mutant and a 240 bp for the wild-type locus. The *Trp53* allele was genotyped with oligonucleotides 5’-CACAAAAACAGGTTAAACCAG-3’ and 5’-AGCACATAGGAGGCAGAGAC-3’ yielding a 370 bp for the mutant and a 288 bp for the wild-type locus. Lastly, we used 5'-CATTGCGTGGACAAT GGCTACTCA-3’, 5'-CTAAGCTTGGCTGGACGTAAACTC-3’ and 5'-GGCAAGTTCCGCGTC ATCC-3' for genotyping *Ctnnb1* by yielding a 300 bp for the mutant and 867 bp for the wild-type locus.

### Tissue dissociation

To generate the organoid models, the mouse lung tissue was cut into 2–3 mm-diameter pieces, and sequentially washed with ice-cold phosphate-buffered saline (PBS) and AdDF3 + (Advanced DMEM/F12 supplemented with 1 × Glutamax, 10 mM HEPES, and 1 × antibiotics/antimycotics). Tissue pieces were dissociated into single cells using prewarmed digestion media containing AdDF3 + , 2% collagenase type II (Thermofisher; 17,101,015), and Y-27632 (Biogems; 1,293,823) for 1 h at 37 °C while mixing every 10 min. When completely digested, collagenase was neutralized by adding 2% Fetal Bovine Serum. After filtering through a 70 μm strainer, cells were washed in AdDF3 + before centrifugation at 500 rcf for 5 min.

### Organoid culture

Cell pellets were resuspended in 65% ice-cold Cultrex Reduced Growth Factor BME, type 2 (R&D systems; 3533- 005–02) and organoid medium, followed by seeding into prewarmed 24-well plates as hanging drops. Following a 15-min incubation in 37 °C 5% CO2, organoid medium overlaying the solidified BME dome was added (media composition provided in Suppl. Table 1). To avoid anoikis, the culture medium was supplemented with Rho kinase inhibitor Y-27632 (10 μM) Biogems; 1293823) for the first 2 days. Organoid growth was monitored every other day starting from day 1 using brightfield imaging (Zeiss AX10 inverted microscope; 3847001274). Organoid medium was changed every 2 days and organoids were passaged every 7 days.

To passage the organoids, the medium was removed, and the organoids were mechanically dissociated, and resuspended in ice-cold PBS. After washing with ice-cold AdDF3 + , organoids were incubated with 1 ml TrypLE Express (Gibco; 12605–010) for 10 min while mixing every 5 min. Subsequently, 4 ml of AdDF3 + was added and the cell suspension was centrifuged at 500 × g for 3 min. Pellets were resuspended in cold BME (65%) and reseeded at 4500 cells/well. Selectivity for cancerous organoids was achieved by confirming if healthy organoids could grow in the respective organoid media as well. Cancerous organoid medium was deprived of fibroblast growth factors (FGF’s) necessary for healthy organoid growth. In case of organoid lines harboring the *Trp53* mutation, an additional selection via the addition of 5 μM Nutlin-3a (Selleckchem; S8059) to the medium was performed. Moreover, no R-spondin-1 was applied to the medium of the triple-mutated organoids (KPC) included in this study.

### Validation of mutation status

To verify the successful Cre-lox recombination after TAM administration and the concordance between parental tumor and generated organoids, genomic DNA extracted from tissue and organoid was analyzed by PCR with the forward primers: 5’- TAAGGCCTGCTGAAAATGACTGA-3’ and reverse primers 5’-GAATTAGCTGTATCGTCAAGGCG-3’ for *Kras* (88 bp), the forward primer 5′-CATTGCGTGGACAATGGCTACTCA-3′ and reverse primer 5′-GGCAAGTTCCGGTCATCC-3′ for *Ctnnb1* (867 bp: WT and ∼645 bp: Δex3) and the forward primer 5’- CACAAAAACAGGTTAAACCCAG -3’ and reverse primer 5’- GAAGACAGAAAAGGGGAGGG -3’ for *Trp53* (Δex2-10: ~ 600).

To validate by Sanger sequencing, PCR products (Suppl. Table 2) were purified using MultiScreenHTS filter plates (Merck Millipore) and resuspended in MilliQ-purified H2O. Sequencing reactions were done with the BigDye Terminator v1.1 Cycle Sequencing Kit (Applied Biosystems/Thermo Fisher Scientific), precipitated using isopropanol and resuspended in formamide. Sanger sequencing was done on a SeqStudio Genetic Analyzer (Applied Biosystems/Thermo Fisher Scientific). Sequence data analysis was done with SnapGene Viewer version 5.2.4.

### Hematoxylin–Eosin (H&E) staining

Lung tissues and organoids were fixed in 4% paraformaldehyde (PFA) for 48 h and 1 h, respectively Organoids were isolated from BME, leaving only the purified organoids, followed by Histogel (Epredia^TM^ HG-400–012) embedding. Both tissue and organoid samples were dehydrated, embedded with paraffin, and sectioned into 3–4 μm thick slices. A standard hematoxylin–eosin (H&E) staining protocol was followed. Briefly, sections were deparaffinized in xylene and rehydrated in a graded ethanol series. Sections were stained with Mayer’s hematoxylin (HTX) and eosin reagents. Subsequently, sections were dehydrated in a graded ethanol series followed by xylene.

### Immunofluorescence staining

For Immunofluorescence (IF), sections were incubated at 60 °C for 2 h, followed by deparaffinization in xylene and rehydration in a graded ethanol series. Antigen unmasking was performed using heat-induced epitope retrieval (pressure-cooker). Sections were washed, permeabilized with 0.25% Triton for 30 min. (except PD-L1 staining) and blocked in 5% goat serum with 0.1% Triton for 1 h at room temperature. Subsequently, slides were incubated with primary antibodies at 4 °C overnight. After washing in PBST, incubation with secondary antibodies for 2 h at room temperature was performed. Nuclei were counterstained with DAPI. For Caspase-3 detection after drug treatment in 8-well chamber slides (Thermo Scientific - 177402), whole organoids were fixed in 4% PFA for 30 min, followed by permeabilization with 0.3% Triton for 30 min and blocking with 10% goat serum + 0.1% Triton X-100 for 1 h. They were incubated overnight with anti-cleaved caspase-3 antibody, followed by Alexa Fluor 488-conjugated secondary antibody for 2 h at room temperature. The 8-well chambers were removed, and nuclei were counterstained with DAPI. Information on antibodies is provided in Suppl. Table 3. Confocal imaging was performed using the Carl Zeiss AIM-system; 2501000334.

### Transduction of KPC cells

The tumor established by KPC organoids (MLT3) was dissociated into single cells (named as MLT3M2) and cultured using RPMI 1640 medium, 2 mM L-glutamine, 25 mM HEPES (Corning) with 10% fetal bovine serum (Gibco), 100 U/mL penicillin–streptomycin (Gibco) at 37 °C with 5% CO_2_. MLT3M2 cells were seeded in a 6-well plate with 1.5 × 10^5^ cells per well and transduced with lentiviral particles carrying the pHIV-iRFP720-E2A-Luc vector in the presence of polybrene with a final concentration of 8 μg/mL. After cell sorting for iRFP-expressing transduced cells with FACS, further validation was performed based on in vitro 2D bioluminescence imaging (BLI) to evaluate the luciferase activity of serially diluted transduced MLT3M2-iRFP-Luc cells using an optical imaging system named In Vivo Imaging System Spectrum computed tomography (CT) (IVIS spectrum CT) (PerkinElmer, Waltham, MA, USA).

### In vivo study

All animal experimental procedures were approved by the Malmö and Lund Animal Ethics Committee (Approval no. 5.8.18–21,851/2022; Sweden). For skin xenograft, healthy, KP, KPC and KPC-LM organoids were harvested and separated from BME. Subsequently, those organoids were resuspended in 30% BME/PBS and subcutaneously transplanted into both flanks of NMRI-nu immunodeficient mice anesthetized with 3% isoflurane gas. Tumor volume and body weight were monitored every two weeks. For orthotopic model, the experiment was performed in female NMRI-nu immunodeficient mice. Prior to the intratracheal tumor transplantation, the animals were anesthetized with a mixture of ketamine (90 mg/kg) and xylazine (10 mg/kg) intraperitoneally (i.p.) and later the mice were fixed on an intubation platform. MLT3M2-iRFP-Luc cells (2.5 × 10^6^) were transplanted intratracheally and two weeks post-injection, the tumor growth was monitored by non-invasive 2D BLI imaging, using IVIS spectrum CT (PerkinElmer, Waltham, MA, USA). Briefly, the mice were anesthetized with 3% isoflurane gas and injected subcutaneously with 150 mg D-Luciferin/kg body weight in PBS prior to imaging. Acquisition of 2D images was taken sequentially with three intervals between different segments of exposures (Emission: open filter, f/stop: 1, binding: 8). The BLI signal intensity was quantified based on the average radiance (photons/s/cm^2^/sr) after deducting the average background signal from the ROI measurement using the Live Image Analysis Software (PerkinElmer, Waltham, MA, USA).

### Drug screening

Kinase inhibitors (Suppl. Table 4) and DNA methyltransferase (DNMT) inhibitors [16] (Suppl. Table 5) were generously provided by Dr. Kazi Uddin. First, 1 μM of kinase inhibitors were applied for broad screening on healthy and KP organoids for five days. This initial screening facilitated the identification of compounds selectively toxic to cancerous KP organoids. Subsequent in-depth screening was conducted with these selected drugs, targeting KP, KPC, and KPC-LM organoids for further evaluation. To conduct drug screening, the organoids were dissociated into single cells, counted, and seeded in 96-well plates at a concentration of 750 cells per well. After 48 h of incubation, organoids were treated for 5 days after which cell viability was determined using the CellTiter Glo 3D viability assay. Briefly, 25 μl of CellTiter Glo reagent (Promega; G9683) was added to the organoid medium while disrupting the BME dome. After 30 min of incubation at room temperature, luminescence was measured at 560 nm. Drug response curves and IC_5O_ values were determined using GraphPad prism 9.5.1.

### Image segmentation

Brightfield microscopy images of Healthy and KP organoids were processed using ImageJ v1.53C. Using a custom-written macros script, images were serially loaded and smoothed using a Gaussian Blur (sigma = 5) and a bandpass filter (3–40 pixels) to posteriorly convert them to a binary mask. These parameters were determined heuristically (verifying visually that the masks resembled the raw images) and applied uniformly across all images to ensure reproducibility and reduce user bias. A watershed algorithm was applied to separate touching objects in the mask. Particles with sizes > 180 pixels and circularity between 0.60–1.00 were analyzed to extract organoids’ shape features. To assess drug efficacy, the average area of all organoids within an image was normalized by the average area from control images. The density was assessed as the number of organoids in the image divided by the average number of control organoids.

### Classification

Normalized area and density values from cancer organoids treated with different drugs and concentrations were used to identify the boundaries that defined clusters corresponding to drug efficacy scores. High inhibition was classified with a score of 3 when Area < 60 and Density < 60. A score of 2 was assigned for Area values between 60 and 80. Low inhibition was represented by a score of 1 for Area ≥ 80, except when Area ranged between 80 and 100 and Density < 61, in which case a score of 2 was assigned.

### Synergy calculation

We combined the targeted drugs (amuvatinib, selumetinib, and midostaurin) with DNMT inhibitor – decitabine. Synergy calculations were performed using the online tool SynergyFinder 3.0, applying both Bliss and HSA models. A synergy score below -10 indicates a likely antagonistic interaction between the two drugs; a score between -10 and 10 suggests an additive interaction; and a score greater than 10 signifies a likely synergistic interaction [17].

### Annexin V-7AAD staining

Apoptosis was evaluated using the Annexin V-FITC Apoptosis Detection Kit (BioLegend - 640922), following the manufacturer's protocol. Briefly, KP and KPC organoids were treated with amuvatinib, selumetinib, midostaurin, and decitabine for four days. Following treatment, the organoids were mechanically dissociated and resuspended in ice-cold PBS. After two washing steps, they were further dissociated using Trypsin/EDTA. The resulting cells were washed twice with ice-cold PBS and resuspended in 100 μl of Annexin binding buffer (~ 1 × 10^5^ cells/ml). Next, 5 μl of Annexin V-FITC and 5 μl of 7-AAD were added, and the samples were incubated for 15 min in the dark at room temperature. Finally, 300 μl of binding buffer was added, and the samples were analyzed using a BD FACS Melody™ flow cytometer. Data were processed using FlowJo v10 software.

### Cell cycle analysis

The cell cycle profile of live cells was analyzed using 7-AAD/saponin staining. KP and KPC organoids were treated with amuvatinib, selumetinib, midostaurin, and decitabine for four days. Following treatment, organoids were mechanically dissociated, resuspended in ice-cold PBS, and washed twice. They were then further dissociated using Trypsin/EDTA. For staining, cells were washed once with PBS and resuspended in 7-AAD/Saponin solution (0.03% Saponin, 25 µg/ml 7-AAD, 1% BSA in PBS). Samples were incubated at 37 °C for 30–60 min. Following incubation, cell cycle distribution was analyzed using a BD FACS Melody™ flow cytometer, and data were processed using FlowJo v10 software.

### Statistical analysis

Non-linear regression analysis and IC_50_ determination were performed using GraphPad Prism 9.5.1.

## Results

### Induction of lung adenocarcinoma in transgenic animals

**Fig. 1:**
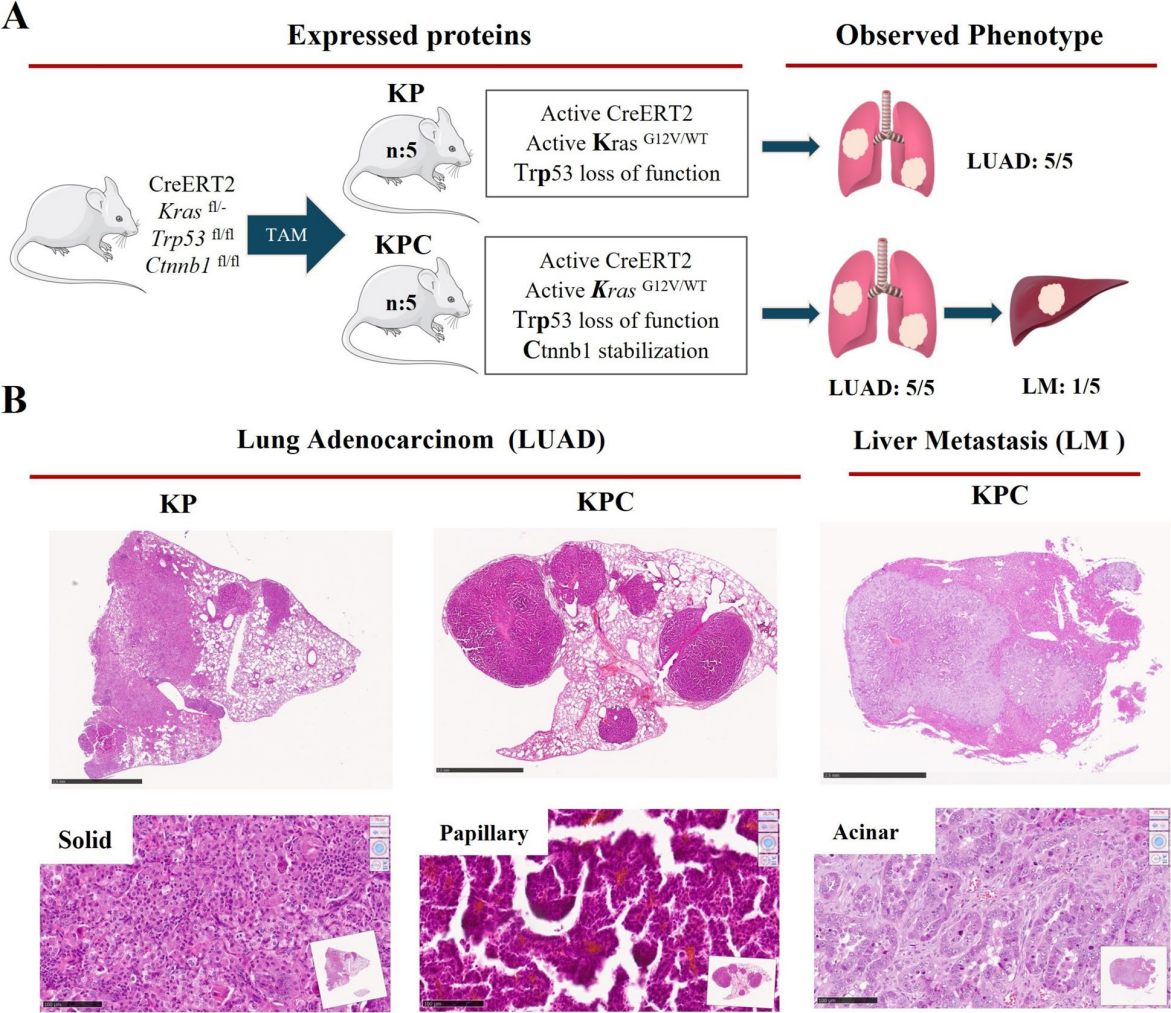
Expression of oncogenic *Kras*^G12V^, inactivation of *Trp*53 and stabilization of *Ctnnb1* promotes tumorigenesis in KP and KPC mice. **A** Schematic representation of tamoxifen (TAM) induced KP and KPC mouse models. **B** Representative images of H&E staining of paraffin sections from lungs in KP, KPC, and liver metastasis in KPC including different growth patterns (papillary, acinar, solid). Scale bars represent 2.5 mm in full size (1x) images and 100 μM in 25 × images, respectively. KP (*n* = 5) and KPC (*n* = 5)

We used KP transgenic animals to initiate lung tumor development. The KP mouse was crossed with *Ctnnb1*^Δex3^to generate KPC model, which harbors mutations in the *Kras*, *Trp53*, and *Ctnnb1* (Fig. 1A). In both the KP and KPC mouse models, TAM injection induces the conditional expression of oncogenic *Kras*^G12V^, which is co-expressed with the β-Geo lacZ reporter gene. This process concurrently inactivates *Trp53* tumor suppressor function and induces stabilization of *Ctnnb1*. After 6 months of TAM administration, we observed the generation of LUAD in both KP and KPC models. Notably, a case of liver metastasis was detected in one of the KPC models (Fig. 1A and Suppl. Figure 1). Both KP and KPC models presented several rounded nodules. Morphologically, all nodules across the cases were non-mucinous adenocarcinomas, displaying a mix of papillary, acinar, and/or solid growth patterns, often featuring compact papillary structures. There were no nodules with lepidic, micropapillary, or mucinous growth patterns. The tumor cells ranged from cuboidal to low cylindrical in solid growth areas, and from low to moderately high cylindrical in and acinar growth areas (Fig. 1B). This detailed morphological assessment highlights the heterogeneity and distinct histological features of LUAD in the KP and KPC mouse models, providing a robust preclinical platform for further studies on tumor biology and therapeutic interventions.

### Establishment of lung adenocarcinoma organoids

**Fig. 2:**
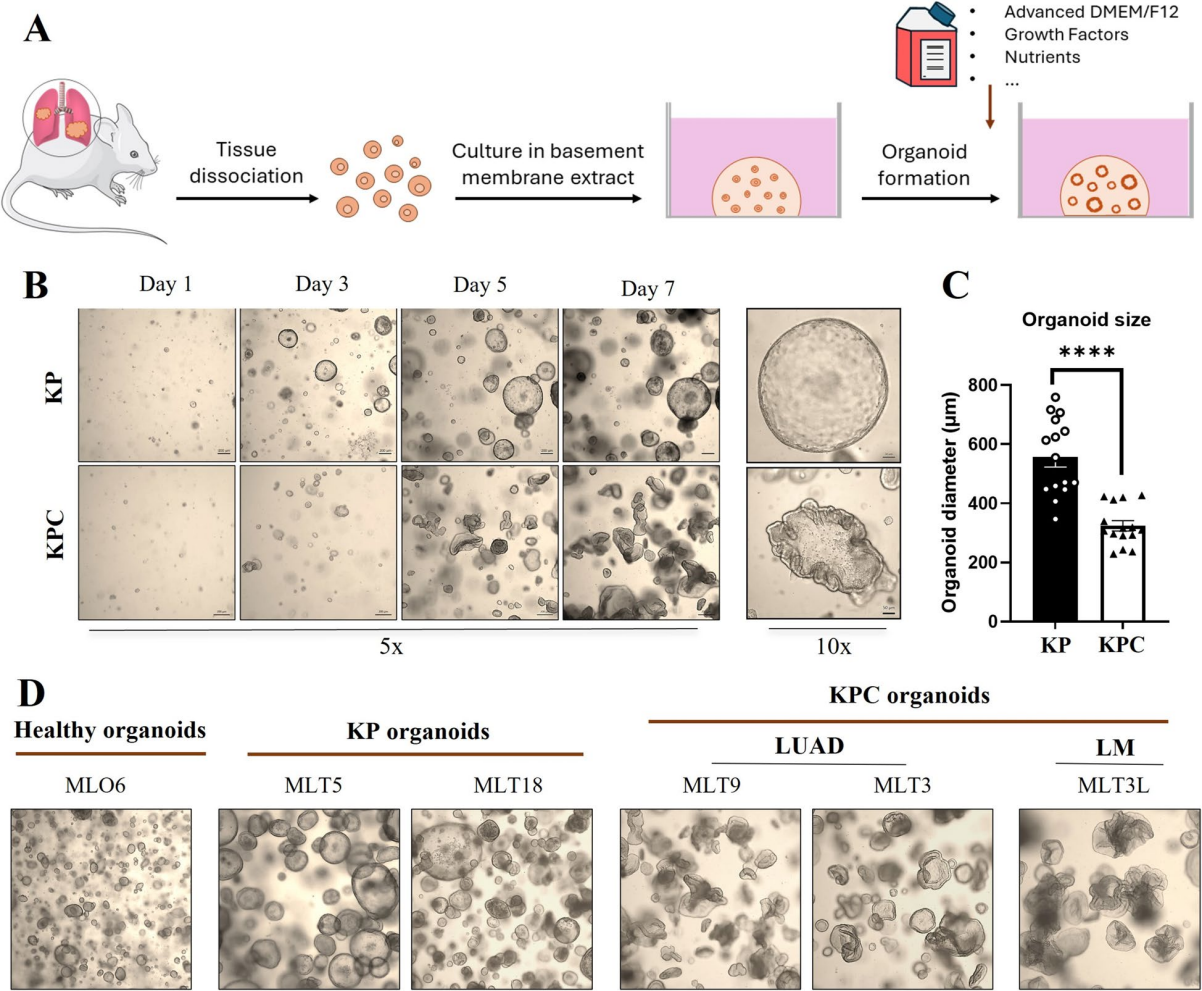
Establishment of organoids from murine healthy lung, LUAD, and LM tissues. Murine lung and liver metastatic tissues were collected to generate organoids based on relevant mutations. **A**) Schematic for organoid generation from primary tissues. **B**) One-week expansion of the KP and KPC organoid models (5X) with scale bars; 200 μm. and their morphology (10X) with scale bars; 50 μm. **C**) Comparison of average organoid diameter between models (*n* = 15 per group). ****p ≤ 0.0001. D) Representative images from healthy organoids. 2 biological replicants of KP organoids (MLT5 and MLT 18), 2 biological replicates of KPC organoids (MLT3 and MLT9) and metastatic liver organoid (MLT3L)

To establish organoid cultures from lung tumor tissue, isolated tumor cells were embedded in BME and overlaid with the appropriate organoid culture medium (Fig. 2A). The organoid lines derived from KP and KPC models were expanded successfully within one week and could be passaged consistently for over ten weeks. The organoids derived from KP models exhibited rounded morphology, while the inclusion of the *Ctnnb1* mutation resulted in highly disorganized and irregularly shaped organoids in the KPC model (Fig. 2B). This distinct morphology was maintained throughout successive passages. The size of organoid size was influenced by the mutational background, with organoids harboring mutated *Ctnnb1* being smaller in diameter compared to other models (Fig. 2C). Healthy organoids exhibited a round shape and liver metastatic organoids, KPC-LM, consistently mirrored the morphology of KPC organoids (Fig. 2D).

The different mutational backgrounds necessitated distinct medium compositions for optimal growth (Suppl. Table 1). Due to oncogenic Kras activation, FGFs were unnecessary for the generation of cancerous organoids. For organoids with mutated Trp53, the medium was supplemented with Nutlin-3a to selectively culture cancerous cells. Additionally, due to the active nuclear stabilization of β-catenin, R-spondin1 was not required for the growth of KPC and KPC-LM organoids, unlike other models. Healthy lung organoids required a complete organoid medium supplemented with growth factors (FGFs) and activators (R-spondin1). However, when the medium for healthy organoids was switched to the KP or KPC medium used for the cancerous organoids, the growth was impaired (Suppl. Figure 2A). These findings indicate that the organoid populations derived from KP and KPC models consisted of exclusively cancerous organoids. (Suppl. Figure 2B).

### KP and KPC organoids retain the genomic and histopathological features of their parental tumors

**Fig. 3:**
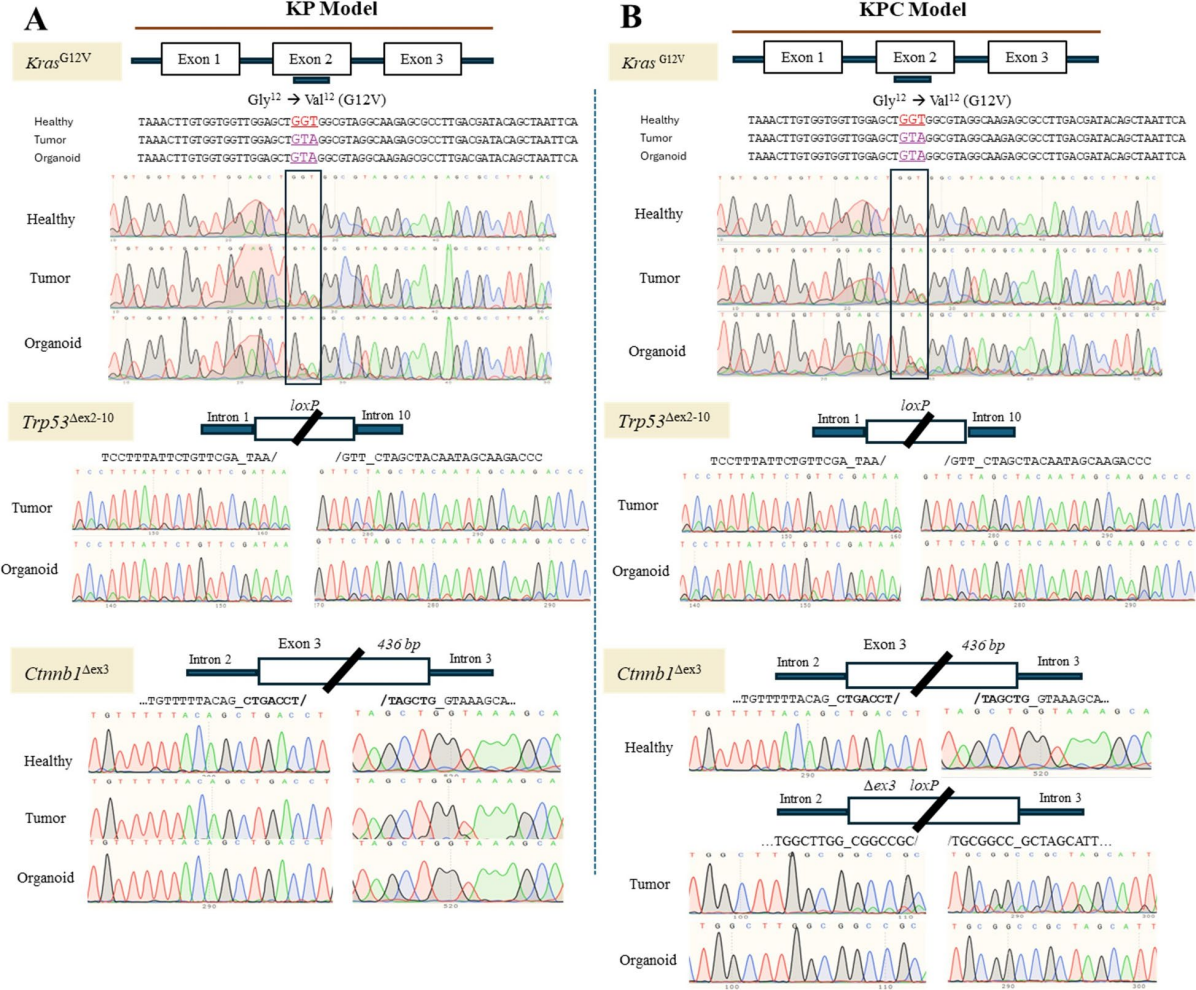
Validation of mutation status in organoids and parental tumors by Sanger sequencing. (**A**) The KP model show a double-mutant with confirmed presence of the *Kras*^G12V^ and *Trp53*^∆ex2−10^ mutations in both tumor and organoid samples of KP model. (**B**) The KPC model shows a triple-mutant with confirmed presence of the *Kras*^G12V^, *Trp53*^∆ex2−10^ and *Ctnnb1*^∆ex3^ mutations in both tumor and organoid samples. Healthy tissue was included as a control and yielded products for the *Kras* and *Ctnnb1* amplicons

To confirm that the organoids retained the specific mutations of parental tumors, we performed Sanger sequencing on both the parental tumor tissues and derived organoids. Mutation status for *Ctnnb1*, *Kras*, and *Trp53* was compared across all organoids, their corresponding original tumor tissues and normal tissue controls (Suppl. Table 2). Sanger sequencing confirmed that the expected mutations were present in both the tumors and the organoids (Fig. 3 and Suppl. Figure 3), demonstrating that the organoids accurately maintained the designed genetic alterations of the parental tissues.

**Fig. 4:**
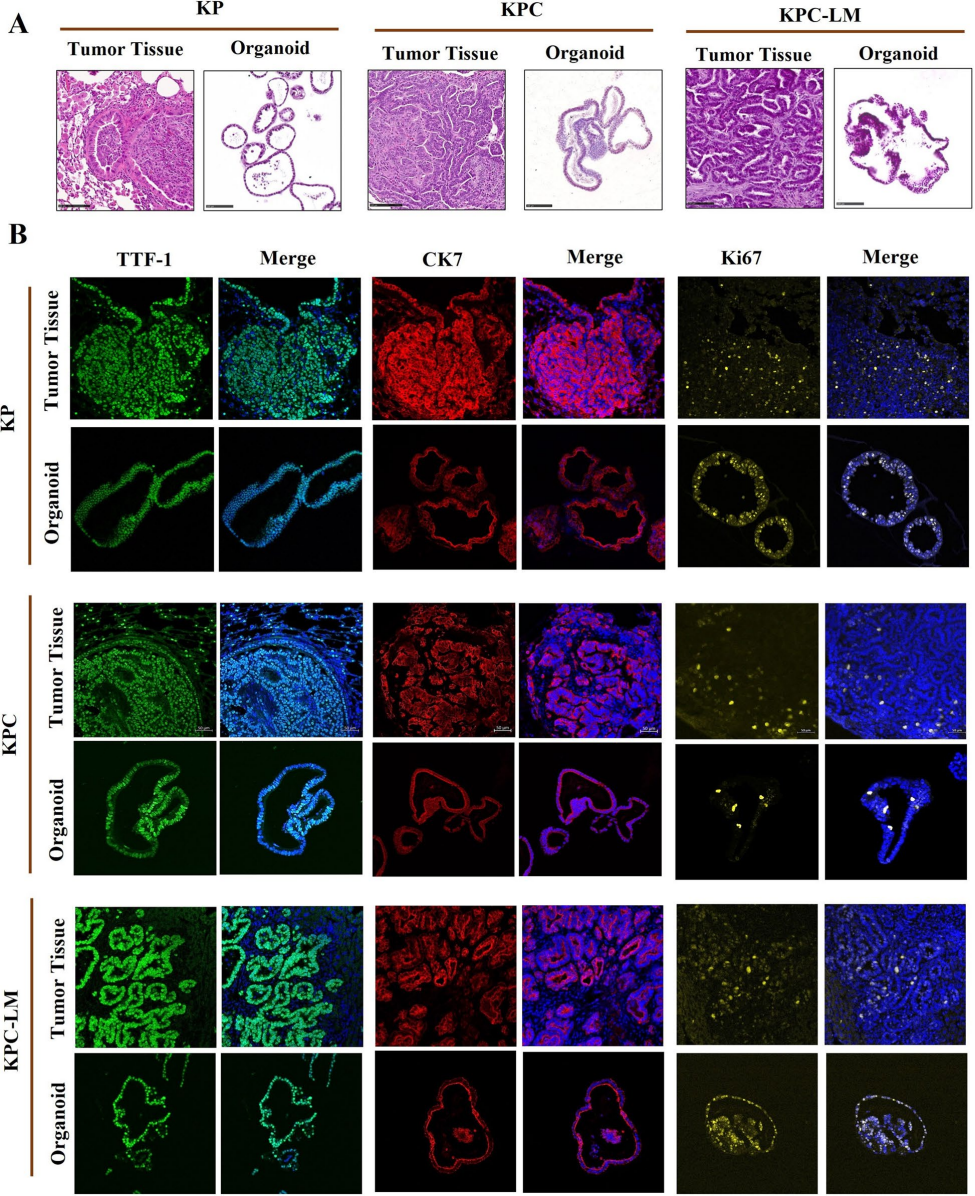
Organoids preserve the histopathological features of parental tumors. (**A**) Representative H&E staining of KP, KPC, and KPC-LM tumors together with H&E staining of the organoids. Scale bar, 100 μm. H&E; hematoxylin & eosin. **B**) Histopathological characterization of murine tissue and organoids by LUAD nuclear marker TTF-1 and cytoplasmic marker CK7, and proliferation marker Ki67. Representative images show the results of immunofluorescence staining. TTF-1, CK7 and Ki67 expression are shown individually and merged with DAPI (blue). TTF1; Thyroid transcription factor-1, CK7; Cytokeratin 7, Ki67: Antigen Kiel 67

Next, to assess whether the organoids preserved key histopathological features of their parental cancer tissues, we performed standard H&E and IF staining. Histological evaluation of the KP, KPC, and KPC-LM organoid lines are conducted by comparing H&E images of organoids with corresponding sections from the parental tissues. The characteristic features of LUAD, such as nuclear pleomorphisms, glandular patterns, presence of prominent nucleoli, and a high nuclear-to-cytoplasmic ratio were found to be highly similar between the organoids and their parental tumor tissues (Fig. 4A). The KPC and KPC-LM organoids exhibited a more heterogenous phenotype and disorganized morphology consistent with observation from the brightfield images.

To further characterization of the organoids, IF staining was conducted to confirm the expression of key LUAD markers in the KP, KPC, and KPC-LM models. Thyroid transcription factor-1 (TTF-1) and cytokeratin 7 (CK7) are markers used to diagnose the histological subtype of lung cancer and to distinguish primary LUAD [18]. IF staining revealed that all organoid lines were positive for TTF-1 and CK7 expression, consistent with their parental tumor counterparts, thereby indicating their LUAD identity. The proliferation marker Ki-67, which indicates cells in the active phases of the cell cycle, was expressed throughout both the tumor tissue and the organoids (Fig. 4B). As an additional control, we conducted IF staining for cytokeratin 5 (CK5), an epithelial marker with squamous cell carcinoma, in KP, KPC, and KPC-LM organoids and their parental tumor tissues (Suppl. Figure 4). The absence of CK5 staining confirmed the LUAD origin of generated organoids, distinguishing them from squamous cell carcinoma phenotypes. The immune checkpoint marker PD-L1 (programmed death-ligand 1) was highly expressed in KP organoids and their parental tissues, whereas KPC organoids and corresponding tissues displayed low expression levels. KPC-LM organoids and parental tumors showed minimal to no PD-L1 expression (Suppl. Figure 4). These findings collectively demonstrate that KP and KPC organoids retain the key genomic and histopathological features of their parental tumors, validating their use as reliable preclinical models for studying lung adenocarcinoma and testing therapeutic strategies.

### In vivo* approaches for validating tumor organoids*

**Fig. 5:**
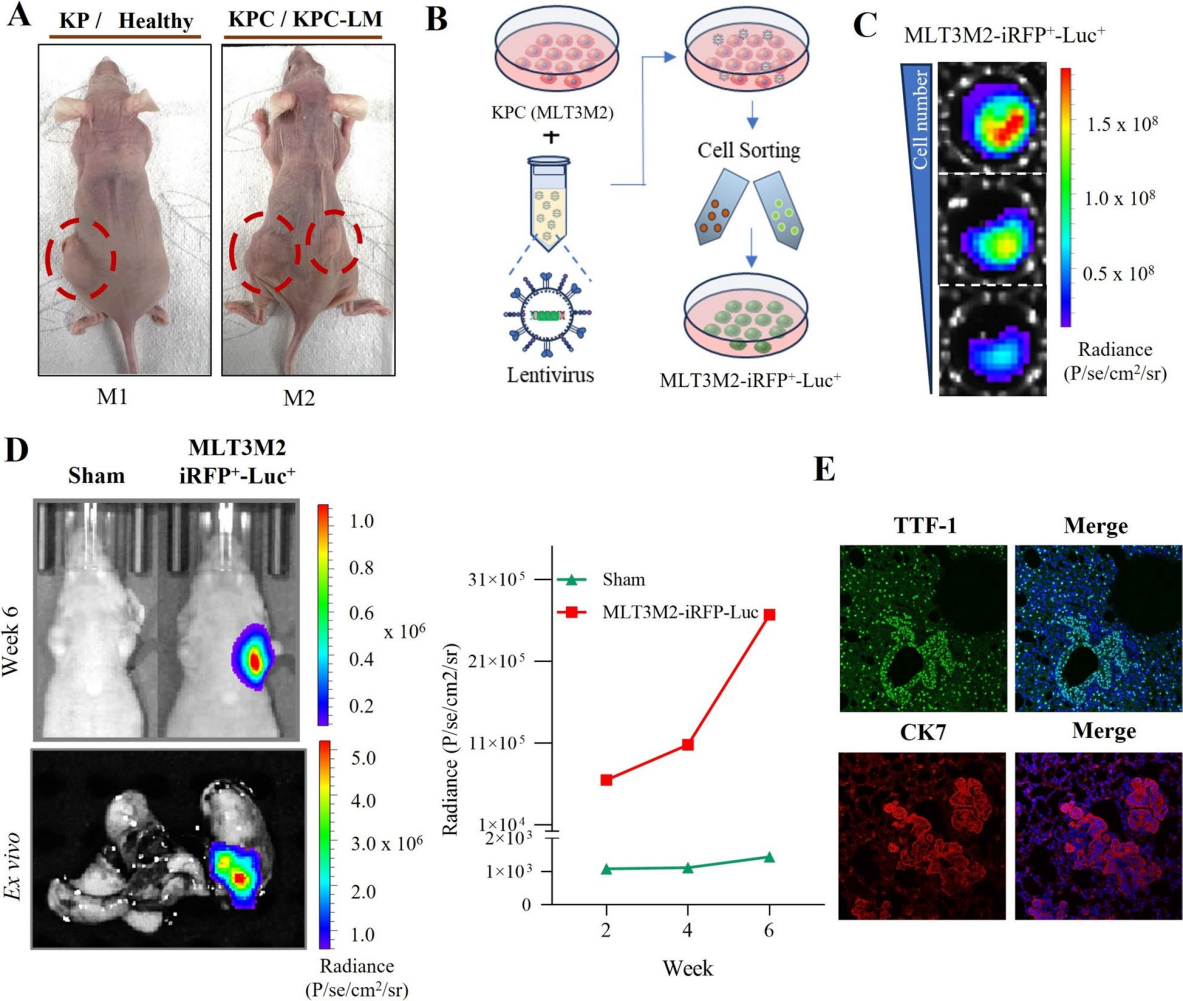
Validation of tumorigenic potential of lung organoids. **A**) Images of mice used to examine the tumorigenic characteristics of organoids. KP/healthy and KPC/KPC-LM organoids were simultaneously transplanted. Red circles indicate the formed tumors. **B**) Schematic for lentivirus transduction to obtain iRFP-Luciferase positive cells. **C**) In vitro evaluation of luciferase activity for serially diluted transduced MLT3M2-iRFP^+^-Luc.^+^ cells using IVIS spectrum. **D**) Visual changes in BLI signal intensity in athymic nude mice following 6 weeks of post-transplantation, ex vivo image of resected lung, and line graph illustrating increase in average radiance overtime. **D**) Representative IF staining images of TTF-1 and CK7 expression individually and merged with DAPI (blue). TTF-1; Thyroid transcription factor-1, CK7; Cytokeratin 7

To assess the tumorigenic capability of KP and KPC organoids, we subcutaneously implanted them into immunocompromised mice. To enhance the likelihood of successful transplantation, we preserved the 3D structures of the organoids, by removing the old BME and without dissociating the organoids. Tumor formation was successfully observed in models transplanted with KP, KPC, and KPC-LM organoids, while no tumor growth was evident in mice transplanted with healthy organoids (Fig. 5A and Suppl. Figure 5A). Thereafter, the tumor generated from KPC organoids-MLT3 was resected and dissociated into single cells (named as MLT3M2) for lentivirus transduction. MLT3M2 cells were cultured and successfully transduced to obtain iRFP-luciferase expressing cells as shown in the schematic (Fig. 5B). Prior to the orthotopic model, the success of transduction in terms of luciferase activity of iRFP + sorted cells were confirmed in vitro (Fig. 5C).

The engraftment potential of KPC organoid—MLT3 was validated in an orthotopic model following intratracheal transplantation of MLT3M2-iRFP-Luc^+^ in immunocompromised nude mice. Over a period of 6 weeks, the overall tumor progression was monitored based on BLI intensity using IVIS spectrum CT once per week. Moreover, ex vivo images of resected lungs were screened after the termination of the study. The results showed an increment of BLI signal quantified as radiance (photons/s/cm^2^/sr) over time (Fig. 5D). The exact location of the tumor engraftment within the lung was confirmed by using 3D BLI (Suppl. Figure 5B). Resected tumor from orthotopic model showed the expression of LUAD markers TTF-1 and CK7 (Fig. 5E).

### Organoid-based drug screening platform to detect selectively targeted drugs and combination treatments against Kras-driven LUAD

Drug repurposing, which involves identifying new therapeutic applications for existing approved drugs or advancing previously studied but unapproved drugs, is a fundamental strategy in drug development [19]. In this study, we conducted a screening of 26 drugs targeting tyrosine kinases (TK), the MAPK pathway, and the PI3K pathway against both healthy and KP organoids (Suppl. Table 4).

**Fig. 6:**
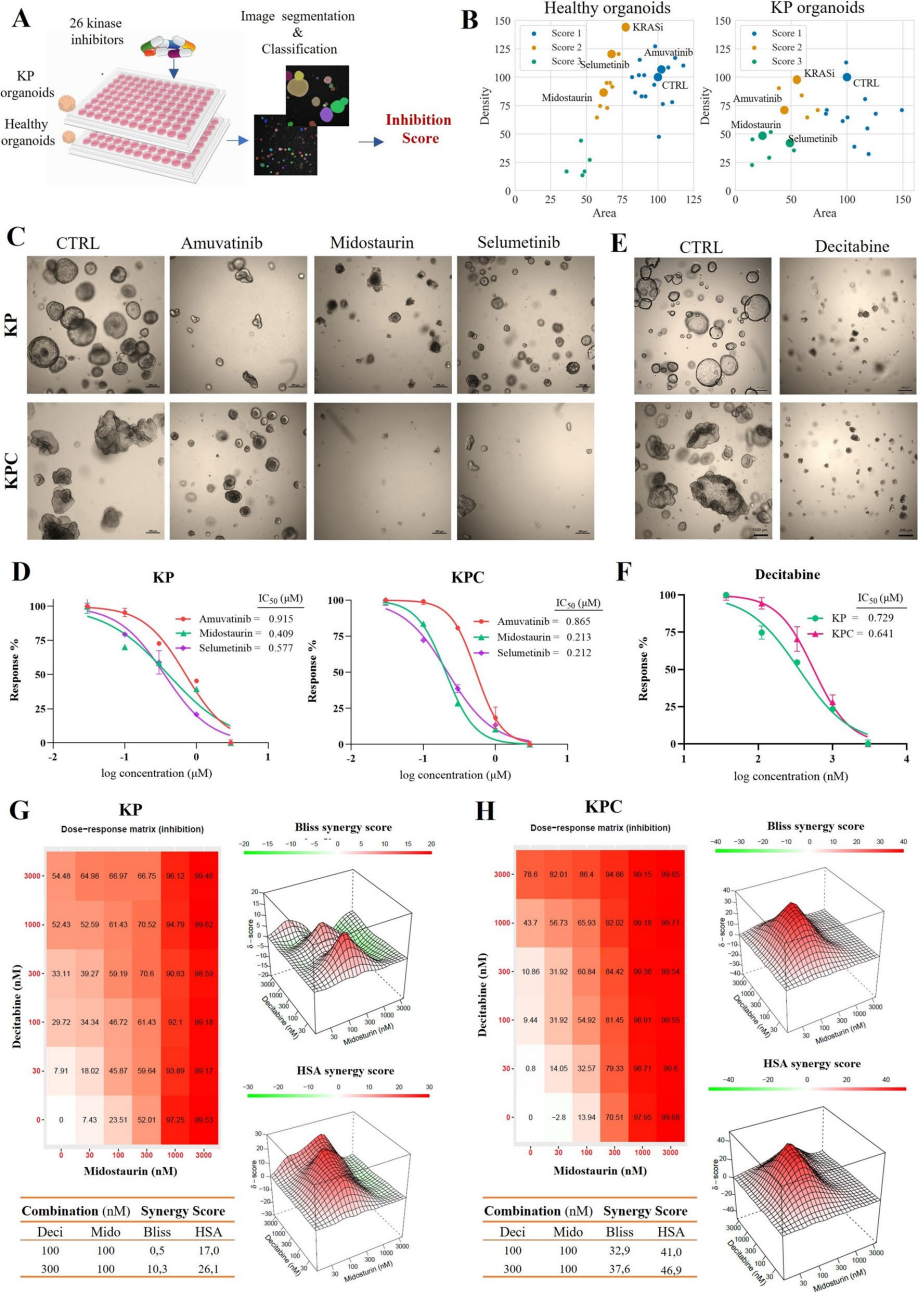
Organoid-based drug screening platform enables the identification of therapeutic agents against *Kras*-driven LUAD. **A**) Schematic showing the workflow of image-based drug screening. **B**) Distribution of values of average area and density ratio for healthy and KP organoids. **C**-**D**) Representative images and dose–response curves for amuvatinib, midostaurin, and selumetinib on KP, and KPC organoids. **E**–**F**) Brightfield images and dose–response curves of KP and KPC mutated organoids after treatment with epigenetic drug- decitabine. IC_50_ is the half-maximal inhibitory concentration of the drug in each model. Data represents the mean ± standard deviation, *n* = 3 technical replicates. **G**-**H**) Combination treatment and synergy analysis of midostaurin with decitabine on KP and KPC organoids. The heatmaps display growth inhibition (%) across different concentration combinations. The tables below the synergy maps present the most synergistic area scores calculated using the Bliss and HSA synergy models.A synergy score between -10 and 10 indicates an additive effect, while scores above 10 suggest synergy. KRASi: KRAS inhibitor, Mido: midostaurin, Deci: decitabine

To assess the effects of these pharmacological treatments, we employed an unbiased analysis on bright field micrography. To ensure objectivity, all images were treated in the same way using batch processing with an in-house macros code in ImageJ (see Methods section) to create masks and identify individual organoids in the pictures. After extracting morphological features from the segmented organoids, the average organoid Area for all treatments was normalized to control values and plotted against the average organoid Density. This analysis revealed three distinct clusters corresponding to different levels of inhibition, determined by predefined thresholds for Area and Density. Using the cluster ID as a proxy for drug efficacy, we referred herein to the classified clusters as the inhibition score for both healthy and KP organoids (Fig. 6A and Suppl. Figure 6). By comparing the inhibition scores, we identified treatments that selectively targeted cancerous organoids, suggesting a promising and safe pharmacological strategy for LUAD. Two multi-targeted tyrosine kinase inhibitors (TKIs), amuvatinib and midostaurin, and one MAPK pathway inhibitor, selumetinib, emerged as effective agents (Fig. 6B).

To further confirm the anticancer efficacy of amuvatinib, midostaurin, and selumetinib, we tested these drugs on KP, KPC (Fig. 6C-D), and KPC-LM organoids(Suppl. Figure 8A). The organoids exhibited diverse sensitivities to these three drug candidates. Midostaurin showed the highest inhibition rate across all organoid types with IC_50_ values of 0.409 μM, 0.213 μM, and 0.394 μM for KP, KPC, and KPC-LM organoids, respectively. Selumetinib followed with IC_50_ values of 0.577 μM, 0.212 μM, and 0.459 μM for KP, KPC, and KPC-LM organoids, respectively. KP organoids were less sensitive to the tested drugs compared to KPC organoids. While KPC-LM organoids demonstrated greater sensitivity than KP organoids, they were more resistant to the tested drugs relative to KPC organoids.

Epigenetic dysregulation plays a key role in many tumor types, including LUAD, through silencing of tumor suppressor genes and activation of oncogenes [20]. A strong association exists between oncogenic KRAS (G12V) signaling and aberrant DNA methylation, leading to the dysregulation of genes involved in critical cancer-related pathways such as DNA repair, cell cycle progression, and proliferation [21, 22]. Here, we investigated the effects of six different DNA methyltransferase inhibitors (DNMTi) on KP and KPC organoids (Fig. 6E-F and Suppl. Figure 7). Among these inhibitors, two are FDA-approved (azacytidine and decitabine), two are currently under clinical trials (5-fluorodeoxycytidine (FdC) and hydralazine), and two are in preclinical stages of development (zebularine and RG108) (Suppl. Table 5). Our results demonstrated that the FDA-approved drug decitabine significantly inhibited the growth of the organoids with IC_50_ values of 0.729 μM and 0.641 μM for KP and KPC organoids, respectively (Fig. 6E-F). FdC, a second-generation DNMTi and decitabine analog, exhibited the most potent inhibitory effect on both KP and KPC organoids with IC_50_ values of 0.592 μM and 0.313 μM, respectively Azacytidine was less effective than decitabine with IC_50_ values of 18.44 μM, 9.04 μM for KP and KPC organoids, respectively. RG108, a second generation DNMTi, inhibited the growth of both KP and KPC organoids (IC_50_: 11.65 μM and 9.26 μM, respectively). Zebularine, a second-generation DNMTi and azacytidine analog, induced growth inhibition exclusively in KPC organoids (IC_50_: 20.2 μM), while Hydralazine showed no significant impact on either KP or KPC organoids (Suppl. Figure 7). These differential responses highlight that KPC organoids are more susceptible to DNMTi treatment compared to KP organoids. The establishment of this organoid-based drug screening platform demonstrates its potential for identifying selectively targeted therapies against LUAD, providing insights into the efficacy of existing drugs, and guiding the development of novel treatment strategies.

Combining epigenetic drugs with other therapies, such as chemotherapy, targeted therapies, and immune-based treatments, has emerged as an attractive strategy for the treatment of cancer [23]. We further evaluated the synergistic potential between the DNMT inhibitor decitabine and kinase inhibitors (amuvatinib, midostaurin, and selumetinib) using both Bliss and HSA models. Low-dose decitabine exhibited strong synergy with midostaurin compared to selumetinib and amuvatinib across KP, and KPC (Fig. 6G-H), and KPC-LM organoids (Suppl. Figure 8B) and KPC-LM organoids. The combination of decitabine and midostaurin consistently demonstrated synergistic effects in all organoid lines.The decitabine and selumetinib combination followed a similar trend, except for KP organoids, where the effect was additive (synergy score < 10) based on the Bliss model (Suppl. Figure 9). The decitabine and amuvatinib combination showed an additive effect in KP organoids, but a highly synergistic response in KPC and KPC-LM organoids (Suppl. Figure 10).

Synergy score maps revealed that the highest synergy was achieved with the combination of 100 nM midostaurin and 30, 100, or 300 nM decitabine. Specifically, in KP organoids, the Bliss model indicated a synergy score of 16.3 for 100 nM midostaurin + 30 nM decitabine, while the HSA model showed a score of 26.1 for 100 nM midostaurin + 300 nM decitabine (Fig. 6G). In KPC organoids, the highest synergy was observed with 100 nM midostaurin + 300 nM decitabine, yielding synergy scores of 37.6 and 46.9 according to the Bliss and HSA models, respectively (Fig. 6H). Similarly, in KPC-LM organoids, the combination of 100 nM midostaurin + 300 nM decitabine produced the highest synergy scores, with 20.4 and 23.8 according to the Bliss and HSA models, respectively (Fig. 8B). The strong synergistic potential of low dose decitabine when combined with various kinase inhibitors highlights its promise as an effective option for combination therapy. Moreover, the increased vulnerability of KPC organoids to combination therapy is likely due to the stabilization of Ctnnb1, suggesting that in cases where Ctnnb1 is stabilized, co-treatment with decitabine could be a particularly effective therapeutic option.

### Midostaurin and decitabine induce cell-cycle arrest and apoptosis in Kras-driven LUAD organoids

**Fig. 7:**
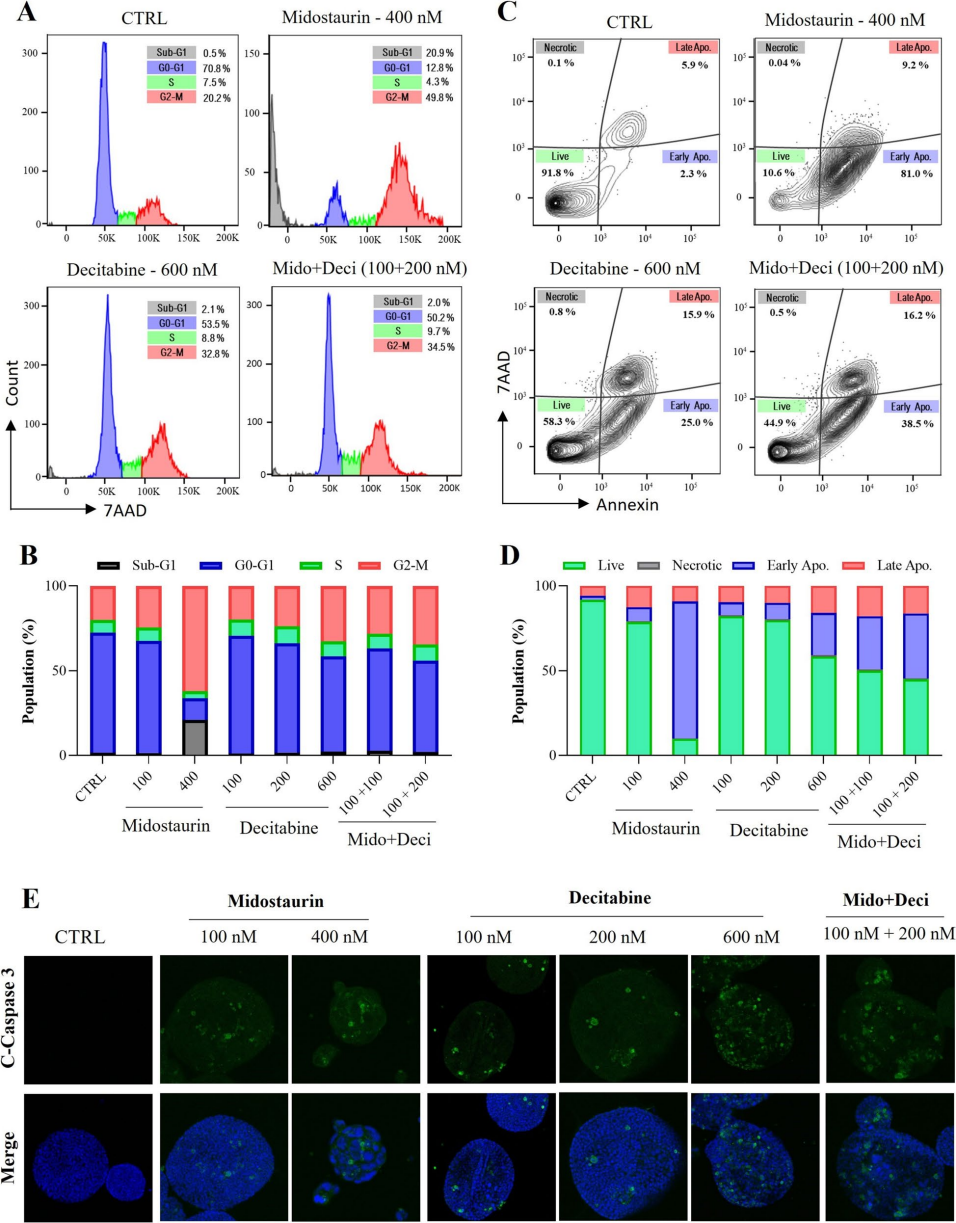
Effect of Midostaurin, Decitabine, and their combination on cell cycle and apoptosis in *Kras*-driven LUAD organoids. **A** Cell cycle distribution of KP organoids analyzed by flow cytometry after treatment with midostaurin, decitabine, and their combination, using 7AAD/saponin staining. **B** Quantitative summary showing the percentage of cells in different phases of the cell cycle. **C** Flow cytometric analysis of cell death in treated KPC organoids stained with Annexin V/7AAD to distinguish live, early apoptotic, late apoptotic, and necrotic populations. **D** Graphical representation (%) of live and apoptotic populations following treatments. **E** Immunofluorescence staining for cleaved caspase to confirm apoptosis induction after drug treatments**.**Mido: midostaurin, Deci: decitabine

Cell growth and death are critical processes in maintaining cellular homeostasis, but cancer cells often disrupt this balance due to dysregulated cell-cycle mechanisms. Inducing cell-cycle arrest at specific checkpoints significantly contributes to antitumor activity [24, 25]. To examine the impact of targeted and epigenetic drugs on cell-cycle progression and apoptosis, we treated KP and KPC organoids with midostaurin, decitabine, and their combinations (Fig. 7A and Suppl. Figures 13–14). Cell-cycle analysis showed that Midostaurin (400 nM) induced strong G2-M arrest (49.8%) and increased the sub-G1 phase (20.9%), indicative of cell death. Combination treatment with lower concentrations of midostaurin and decitabine also effectively induced G2-M arrest (34.5%), supporting our previous synergy findings (Fig. 6G-H and Suppl. Figures 8–10).

We further analyzed apoptosis using Annexin V and 7AAD staining under the same experimental conditions (Fig. 7C-D). Consistently, midostaurin emerged as the most potent inducer of apoptosis, causing over 80% total apoptosis at 400 nM in KPC organoids. Notably, the combination of low-dose decitabine (200 nM) and midostaurin (100 nM) induced approximately 55% apoptosis, surpassing the apoptosis observed with high-dose Decitabine (600 nM, ~ 40%). Similar patterns of apoptosis induction were observed across KP and KPC organoids for all tested drugs (Suppl. Figures 13–14).

To confirm apoptosis induction mechanistically, we performed immunofluorescence staining for cleaved caspase-3, a key executor of apoptosis. Our results confirmed the increased activation of caspase-3 following single and combination drug treatments (Fig. 7E and Suppl. Figure 15).

Overall, these findings clearly demonstrate that midostaurin, alone or in combination with decitabine, effectively disrupts cell-cycle progression and induces apoptosis in KRAS-driven lung adenocarcinoma organoids, highlighting their therapeutic potential.

## Discussion

Kras is a key regulator of cell proliferation, growth, and survival, frequently mutated in multiple cancer types. While Kras inhibitors have marked a breakthrough in targeted therapy, their clinical efficacy is limited by resistance mechanisms, including adaptive signaling activation and secondary mutations [6]. As a result, combination strategies and next-generation inhibitors are needed to enhance therapeutic efficacy and overcome resistance. To address this issue, we generated club cell-initiated LUAD by crossing mice expressing Cre recombinase under the control of the club cell secretory protein promoter (CC10-CreERT2) with mice harboring *Kras* ^G12V^ & *Trp53*^Δex2−10^ (KP) in this study. By further crossing the KP mice with *Ctnnb1*^Δex3^, we established the KPC model, which carries mutations in *Kras*, *Trp53*, and *Ctnnb1*. To the best of our knowledge, this is the first study of a KPC mouse model for LUAD. The generation of LUAD in GEM models provides valuable insights into the pathogenesis and potential therapeutic targets of lung cancer [26]. Rosigkeit et al. reported that club cells act as progenitors for LUAD development as an alternative to alveolar type II(AT2) cells [27].

The concurrent activation of Wnt/β-catenin signaling along with the constitutive expression of a *Kras*^G12D^ in LUAD (CC10-Cre; *Kras*^G12D^; *Ctnnb1*^Δex3^:KC model) has been shown to significantly increase both the number and size of tumors compared to *Kras*^G12D^ expression alone, without metastasis [28]. Notably, in our study, the KPC model demonstrated liver metastasis, emphasizing the aggressive nature of tumors with concurrent *Kras*, *Trp53*, and *Ctnnb1* mutations. This observation aligns with the findings of Fujishita et al. who developed a novel mouse model of colorectal cancer that spontaneously develops liver metastasis, by introducing sporadic mutations of *Kras, Trp53, Ctnnb1*, and *Smad4* (KPCS) genes [29]. In their study, 100% of KPCS mice developed at least one invasive intestinal adenocarcinoma and 23% exhibited liver metastasis. In the same study, KPC mice developed invasive adenocarcinomas and 1 out of 30 KPC mice showed liver metastasis consistent with our findings [29]. Neither the KP nor the KC models exhibited liver metastasis, whereas the KPC model in our study did in line with observations from colorectal cancer studies, suggesting a cooperative interaction between Trp53 and Ctnnb1 in promoting liver metastasis in *Kras*-driven LUAD. These findings highlight the unique metastatic potential associated with the combined mutations in the KPC model, providing a deeper understanding of the molecular mechanisms driving metastasis in LUAD and identifying potential avenues for targeted therapies.

Organoids have emerged as valuable preclinical models due to their ability to closely mimic the genetic and histopathological features of patient tumors, making them more reliable than traditional 2D cultures.. [15]. The organoids offer several advantages, including short culture time, cost-effectiveness, and high construction success rates, making them ideal for drug screening and precision medicine. [30] However, the organoids also have notable limitations. The success rate of organoid establishment varies across tumor types, and the lack of standardized culture protocols leads to inconsistent results. Furthermore, organoids cultures often lack key components of the tumor microenvironment, such as fibroblasts, immune cells, and vascular structures, which are critical for tumor progression and response to immunotherapies. The requirement for specialized media, cytokines, and inhibitors also adds to the cost and complexity of their use [30]. Previously, eighty-four organoids were established from patients with advanced LUAD, including a *KRAS*^G12D^ mutated case [31]. Another study, successfully established NSCLC patient and PDX organoid lines, with a *KRAS* G13C, whereas Naranjo et al. generated *Kras*^G12D^ mutant, *Trp53*-deficient (KP) organoids by modeling Kras alteration with p53 loss in wild-type AT2 organoids, using adenovirus-expressed Cre recombinase (Ad5-Cre) [32]. Among KRAS mutations, *TP53* is one of the most common co-existing mutations with *KRAS* present in almost 50% of cases [9]. Additionally, abnormal activation of β- catenin (CTNNB1^ex3^) has been shown to synergize with *KRAS* to enhance tumor formation [10]. Notably, inhibition of the Wnt pathway has been demonstrated to abolish KRAS^G12V^ -induced migration, indicating that metastasis driven by KRAS^G12V^ is Wnt-dependent [11]. To the best of our knowledge, this is the first study that establishes the *Kras*^G12V^-driven LUAD organoids in combination with both *Trp53* (KP) and *Ctnnb1* (KPC) including a liver metastatic LUAD organoid (KPC-LM).

Morphological analysis revealed the KP organoids generally exhibited round, acinar shape, while the additional Ctnnb1 mutation in KPC organoids resulted in a highly disorganized structure. The irregular shape of the triple mutated organoids can be attributed to their mutational background. β-catenin, a key component of the Wnt-signaling pathway, is profoundly involved in cell–cell interactions. It binds E-cadherin and links adherent junctions to the actin cytoskeleton of the cell. Mutant forms of β-catenin can induce transcription of negative regulators of E-cadherin, disrupting cell–cell interactions and potentially affecting organoid growth potential [33, 34]. Consistent with these findings, it has previously been reported that co-expression of *Ctnnb1*^Δex3^ and K*ras*^G12D^ alters the phenotype of bronchiolar epithelial cells of the lung is associated with decreased E-cadherin expression [28]. Further experiments are needed to evaluate E-cadherin expression and its relationship to mutant β-catenins in the context of LUAD.

Characterization of organoids is crucial to ensures they accurately replicate the physiological, genetic, and histological features of the original tissue, which is essential for their validity in disease modeling and therapeutic testing [35]. Sanger sequencing was conducted to validate the engineered mutations in parental LUAD tissue and derived organoids. This analysis confirmed the successful establishment of the murine model and accurate recapitulation of the parental tumor's mutational background identifying a missense mutation in the *Kras* gene (G12V), a deletion in *Trp53* (Δ2-10), and an exon 3 deletion in *Ctnnb1*, all of which were retained in the organoids. Histologically, the organoids closely mirrored LUAD features, expressing the markers TTF-1 and CK7, but not the squamous marker CK5. IF staining for the immune checkpoint marker PD-L1 (programmed death-ligand 1) revealed strong expression in KP organoids, while liver metastatic KPC organoids and tissues exhibited very weak expression, and KPC-LM showed almost no PD-L1 expression. Immunotherapy has significantly improved outcomes for patients with advanced lung cancer, but studies indicate that monotherapy is largely ineffective for NSCLC patients with liver metastases [36, 37]. Our findings, which highlight the low expression of tumor immune markers in KPC models, may help explain the limited efficacy of immunotherapy in NSCLC liver metastases. The tumorigenic potential of these organoids was validated through skin xenograft in immunocompromised mice, with robust tumor development and successful orthotopic lung cancer modeling using iRFP-luciferase transduced organoids. These findings confirm the capacity of organoids to replicate primary tumor characteristics and their utility in experimental and therapeutic applications.

The establishment of an organoid-based drug screening platform enabled the identification of selectively targeted drugs against cancerous organoids, offering a promising approach for personalized cancer therapy. Through image-based analysis, we conducted broad drug screening, allowing us to effectively narrow down the list of candidates. Epidermal growth factor receptor (EGFR), a receptor tyrosine kinase (RTK) plays a vital role in cell proliferation and migration with most of the signaling occurring at the plasma membrane, stimulating the downstream MAPK and PI3K pathways via KRAS [38]. Among the 26 targeted drugs tested from the TK, MAPK, and PI3K pathways, three drugs amuvatinib, midostaurin, and selumetinib showed selective inhibition of KP organoids relative to healthy ones and more effective than Kras inhibitor-6H05. The combination of MEKi (trametinib) with multityrosine kinase PKC inhibitors (mtPKCi; lestaurtinib and midostaurin) has been identified as an effective therapeutic strategy for a significant subset of mutant *KRAS* LUAD in both in vitro and in vivo [39]. Flemington et al. also reported that in a subset of *KRAS*-mutant NSCLC cell lines, the combination of AZD0364 and selumetinib exhibited high synergy, resulting in more profound and sustained suppression of the RAS/MAPK pathway compared to single-agent treatment [40]. Additionally, amuvatinib combined with βIII-tubulin suppression significantly reduced cell proliferation in NSCLC [41]. We further tested three drug candidates, amuvatinib, midostaurin, and selumetinib, on various organoid lines (including KP, KPC, and KPC-LM). The results showed different sensitivities to the drugs among the organoid lines. Midostaurin demonstrated the highest inhibition rate. Notably, KP organoids were less vulnerable compared to KPC and KPC-LM organoids. This variation highlights the importance of considering genetic differences when selecting targeted therapies and adjusting the treatment dosage.

Cancer epigenetics refers to the study of heritable changes in gene expression that occur without altering the underlying DNA sequence, playing a critical role in cancer development and progression. These epigenetic changes can include DNA methylation, histone modifications, and regulation by non-coding RNAs [42, 43]. DNA methylation is catalyzed by a group of enzymes called DNA methyltransferases (DNMTs) and inhibitors against these enzymes can activate silenced genes at low doses and cause cytotoxicity at high doses. The ability of DNMT inhibitors to reverse epimutations is the basis of their use in novel strategies for cancer therapy [44]. One example is that DNMT inhibitor, decitabine, can reverse the hypermethylation status of EGFR promoters in different cancer types by enhancing *EGFR* expression and reversing EGFR-TKI resistance [45]. In a case report, Han et al. reported beneficial results of 3 patients with advanced NSCLC carrying adverse immune checkpoint inhibitor (ICI) biomarkers, such as low tumor mutational burden. Surprisingly, all three patients responded well to low-dose decitabine combined with camelizumab, with slight adverse events, indicating that low-dose decitabine can sensitize ICIs [46]. In this study, we investigated the efficacy of DNMT inhibitors including decitabine against *Kras*-driven LUAD organoids. Consistent with their response to kinase inhibitors, KP organoids demonstrated lower sensitivity to DNMT inhibitors compared to KPC organoids. Decitabine and its second-generation analog, FdC, exhibited the highest efficacy on both KP and KPC organoids compared to other DNMT inhibitors.

Combination cancer therapies are designed to enhance the effectiveness and strength of treatment responses while minimizing the risk of patients developing acquired resistance [47]. Here, we further evaluated decitabine in combination with selected kinase inhibitors. The results demonstrated that low-dose decitabine, in particular, showed a strong synergistic effect when combined with midostaurin, followed by selumetinib and amuvatinib. It appears that the presence of the *Ctnnb1* mutation in KPC organoids renders these cells to be more sensitive to the combination therapy with low-dose decitabine. Our findings further demonstrate that midostaurin and decitabine effectively disrupt cell-cycle progression and induce apoptosis in *Kras*-driven LUAD organoids. Midostaurin induced strong G2-M arrest and increased the sub-G1 population, while its combination with decitabine at lower doses also significantly promoted cell-cycle arrest. Annexin V/7AAD staining confirmed midostaurin as a potent apoptosis inducer, with combination treatment achieving greater apoptosis than high-dose decitabine alone. Increased cleaved Caspase-3 expression further validated apoptotic activation. These results highlight the therapeutic potential of midostaurin, alone or in combination with decitabine, for *Kras*-driven LUAD.

In this study, we successfully established K*ras*-mutant LUAD organoids and conducted a comprehensive characterization to identify potential therapeutic candidates. Our findings highlight the efficacy of targeted and epigenetic therapies, including a DNMT inhibitor-based combination strategy, as a promising approach for *KRAS*-mutant LUAD. These results provide valuable insights into therapeutic vulnerabilities in this challenging lung cancer subtype, supporting further investigation into novel treatment strategies.

## Supplementary Information

Supplementary Material 1.

## Data Availability

All the data generated or analyzed during this study are included in this published article and its supplementary files.

## Abbreviations

KRAS: Kirsten Rat Sarcoma Viral Oncogene Homolog
LUAD: Lung adenocarcinoma
GEM: Genetically engineered mouse
DNMT: DNA methyltransferase
NSCLC: Non-small cell lung cancer
SCC: Squamous cell carcinoma
FDA: Food and Drug Administration
*Scgb1a1*: Secretoglobin Family 1A Member 1
TAM: Injection of tamoxifen
FGF’s: Fibroblast growth factors
H&E: Hematoxylin-Eosin
PFA: Paraformaldehyde
IF: Immunofluorescence
BLI: Bioluminescence imaging
CT: Computed tomography
TTF-1: Thyroid transcription factor-1
CK7: Cytokeratin 7
CK5: Cytokeratin 5
Ki67: Antigen Kiel 67
AT2: Alveolar type II
EGFR: Epidermal growth factor receptor
RTK: Receptor tyrosine kinase
