# Supplementary material for: Advanced organoid models for targeting Kras-driven lung adenocarcinoma in drug discovery and combination therapy
Source: J Exp Clin Cancer Res. 2025 Apr 24;44:128. doi: 10.1186/s13046-025-03385-9 (PMC12020293; doi:10.1186/s13046-025-03385-9)
Supplement: Supplementary file 1 — Supplementary Material 1. [file 13046_2025_3385_MOESM1_ESM.pdf]

Supplementary figures

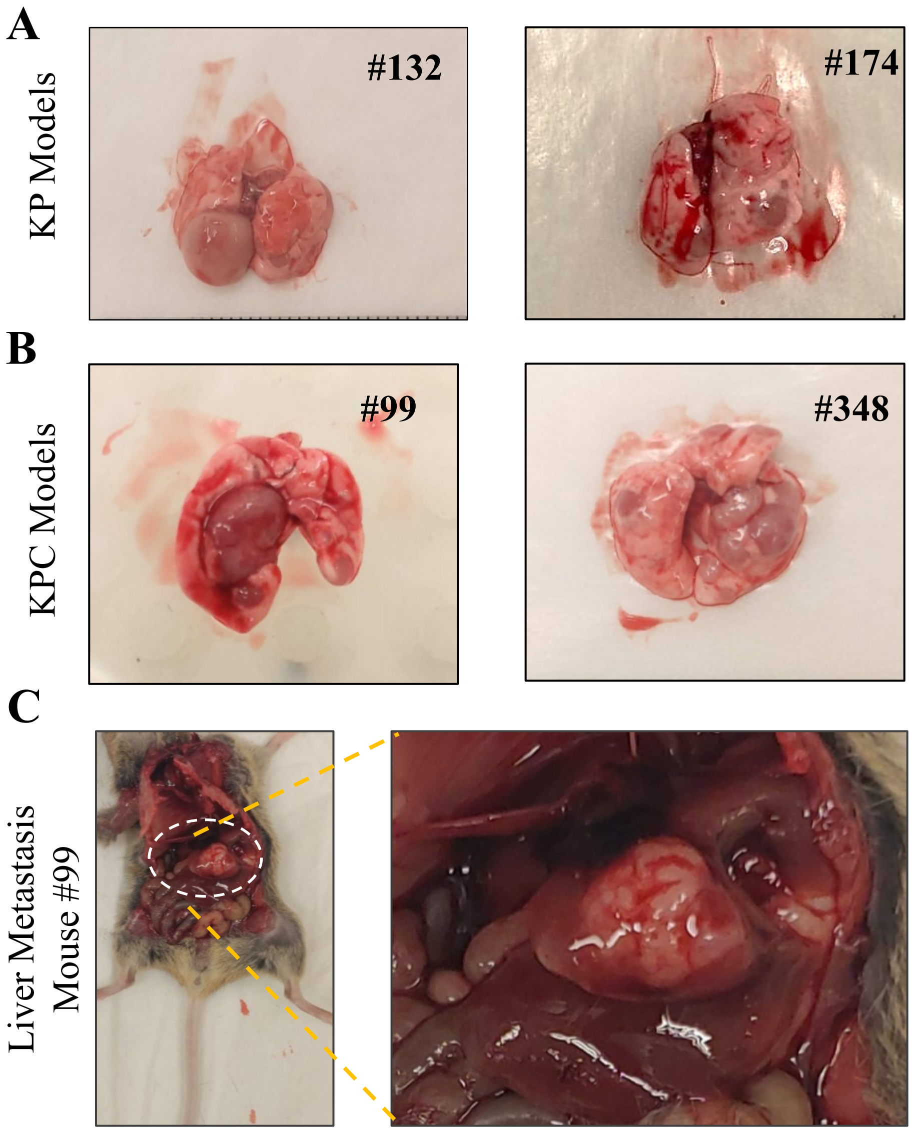

Suppl. Fig. 1: Ex vivo images of resected lungs from (A) KP, (B) KPC models and (C) liver metastasis case.

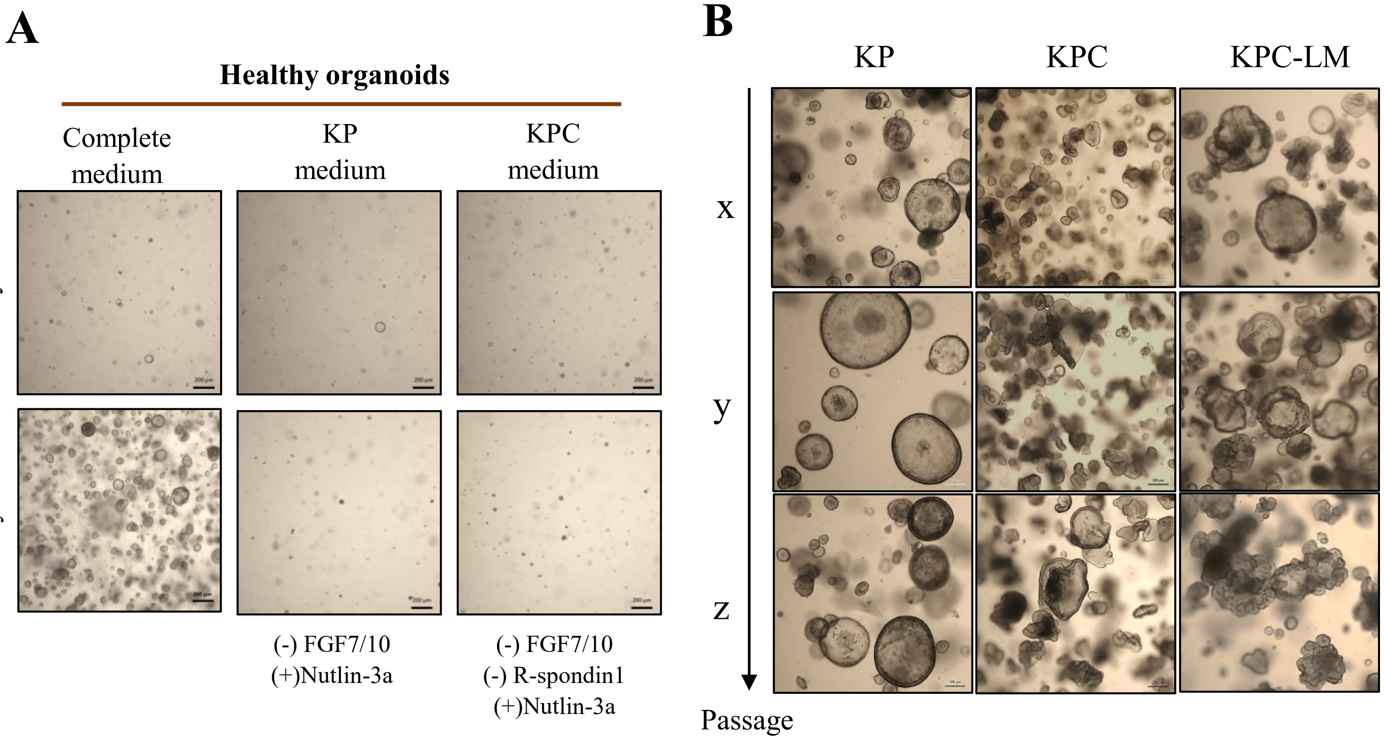

Suppl. Fig. 2: A) Growth of healthy organoids in complete, KP and KPC mediums. B) Organoids retain stable morphology across passages. Day 7 images for each organoid culture are shown. For KP: x = 6, y = 7, z = 9. For KPC: x = 5, y = 7, z = 8. For KPC-LM: x=3, y=4, z=5

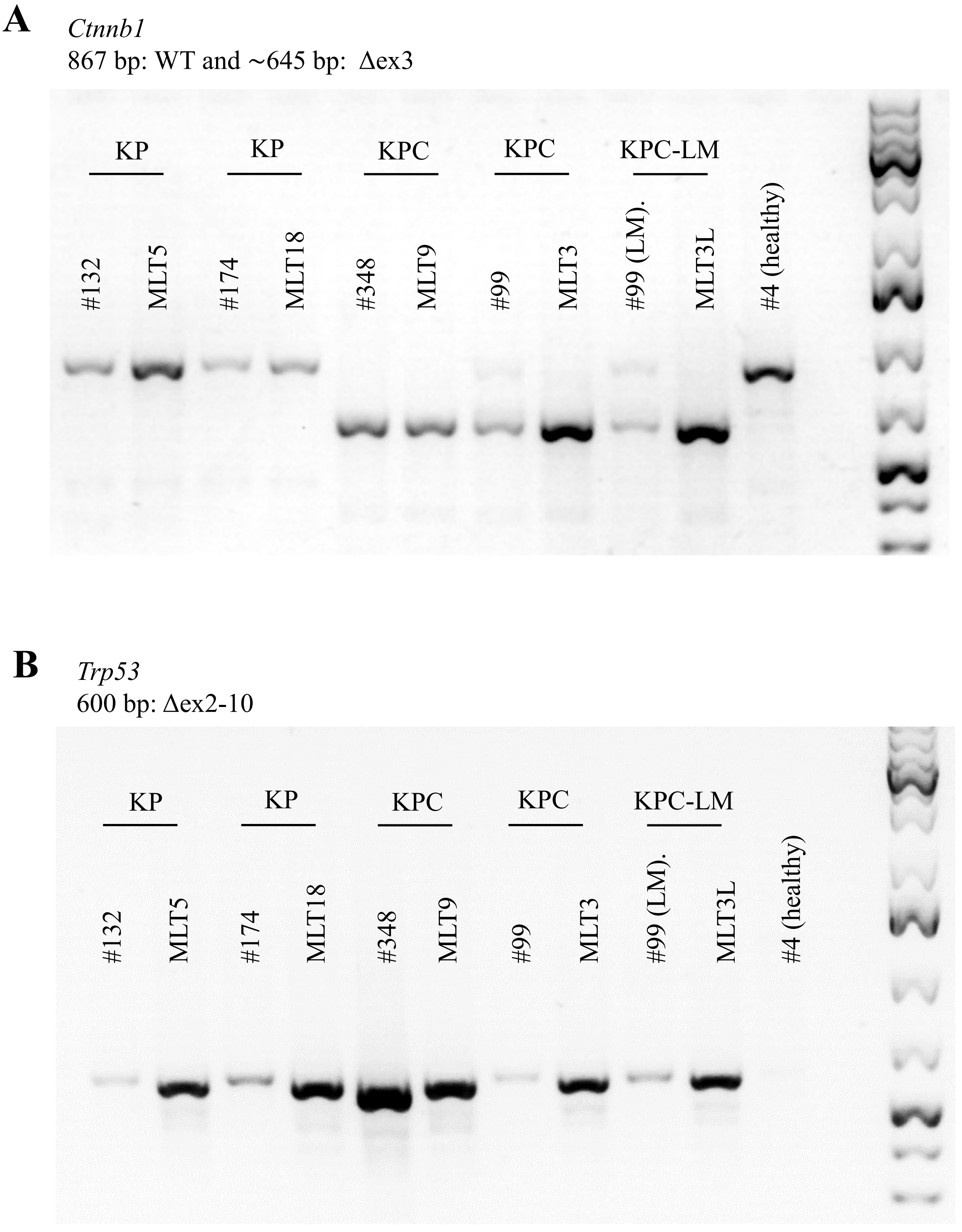

Suppl. Fig. 3: Genotyping analysis of LUAD tumors and organoids (A) *Ctnnb1* and (B) *Trp53*. Numbers with # indicate individual mouse resected tumor, with each tumor sample paired with its corresponding derived organoid (MLT). Liver metastasis (LM) in KPC mouse- #99.

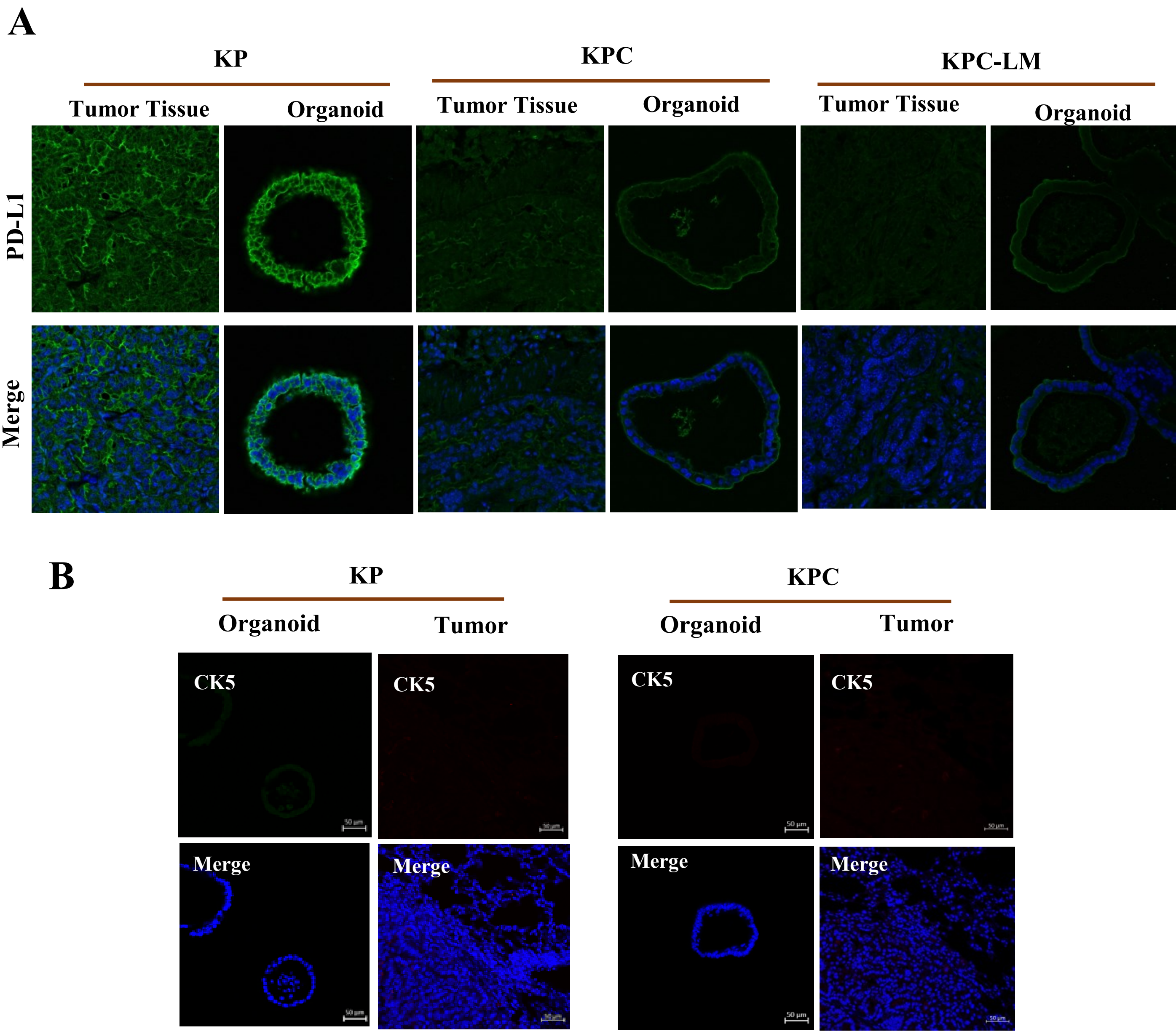

Suppl. Fig. 4: (A) PD-L1expression in KP, KPC and KPC-LM orgnaoids and tissues. (B) CK5 results on KP and KPC organoids and tissues. Scale bar, 50 µm. Lung squamous cell carcinoma marker, CK5, was used as a control. PD-L1: Programmed death-ligand 1; CK5: Cytokeratin 5

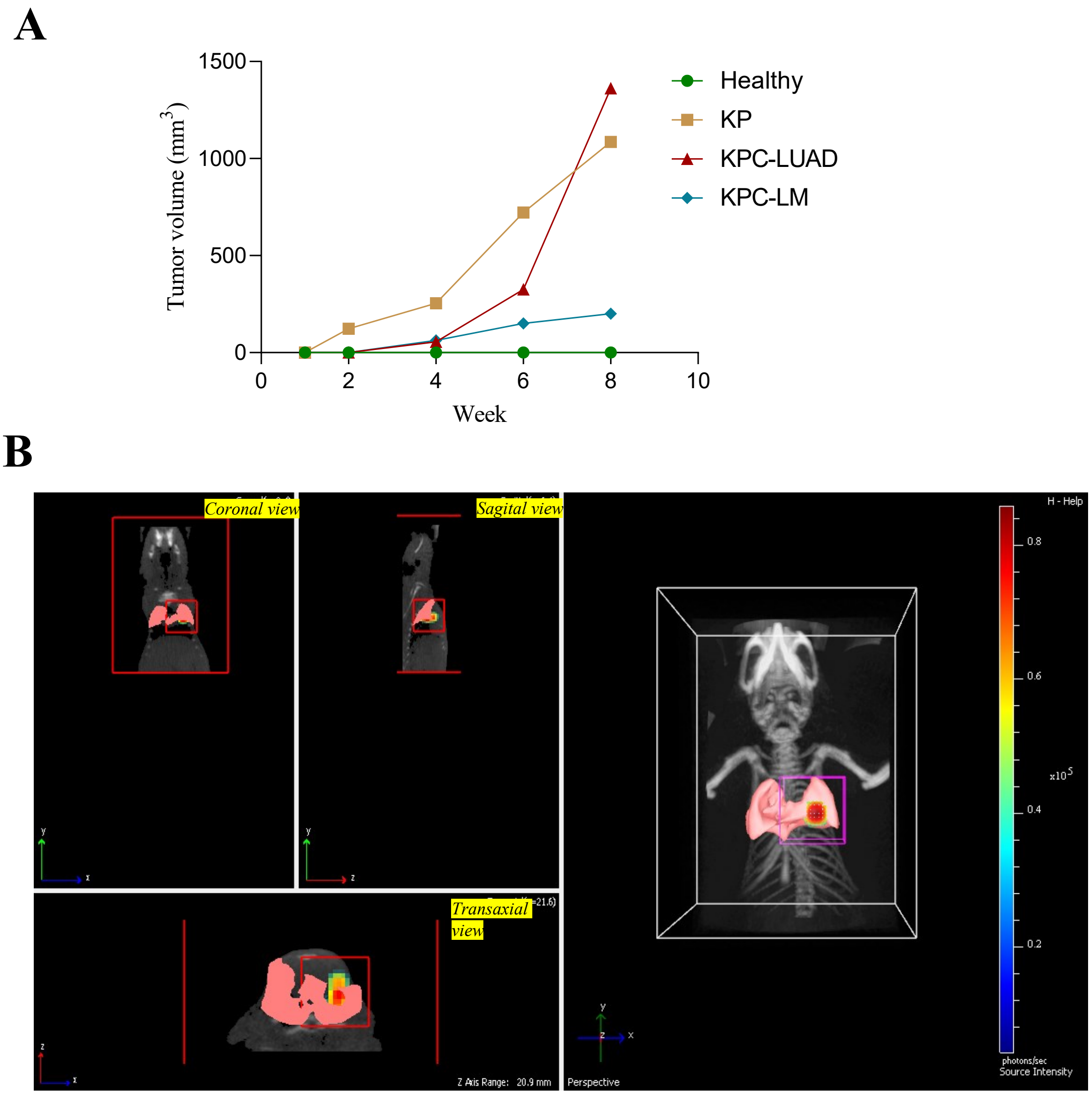

Suppl. Fig. 5: A) Tumor volume result of skin xenografts. B) Ventral side 3D µCT representative images with the mouse organ atlas showing tumor location in the lung after six weeks of MLT3M2-iRFP-Luc orthotopic implantation using Living image analysis software.

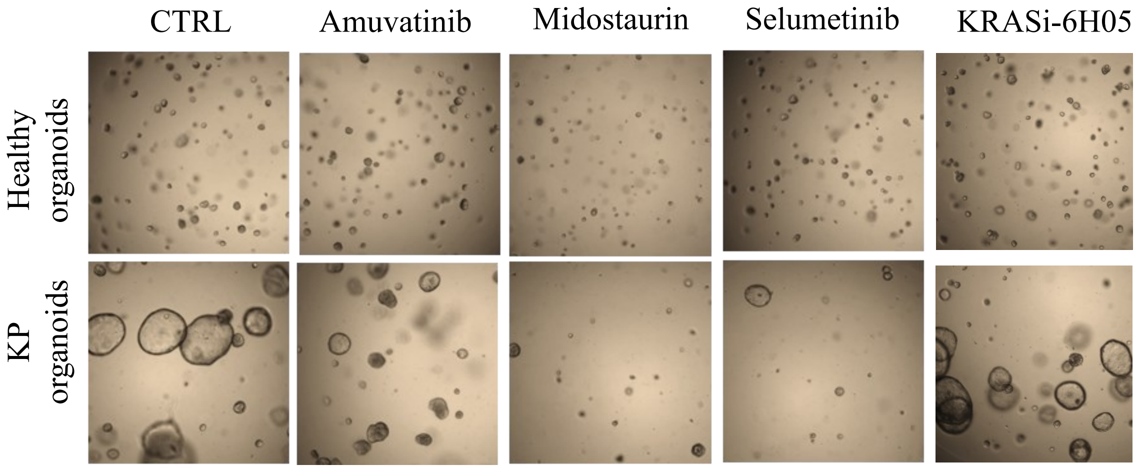

Suppl. Fig. 6. Representative 5X images of healthy and KP organoids after treatment with selected kinase inhibitors – Amuvatinib, Midostaurin, Selumetinib and KRASi-6H05. Images were taken on day 5 of treatment.

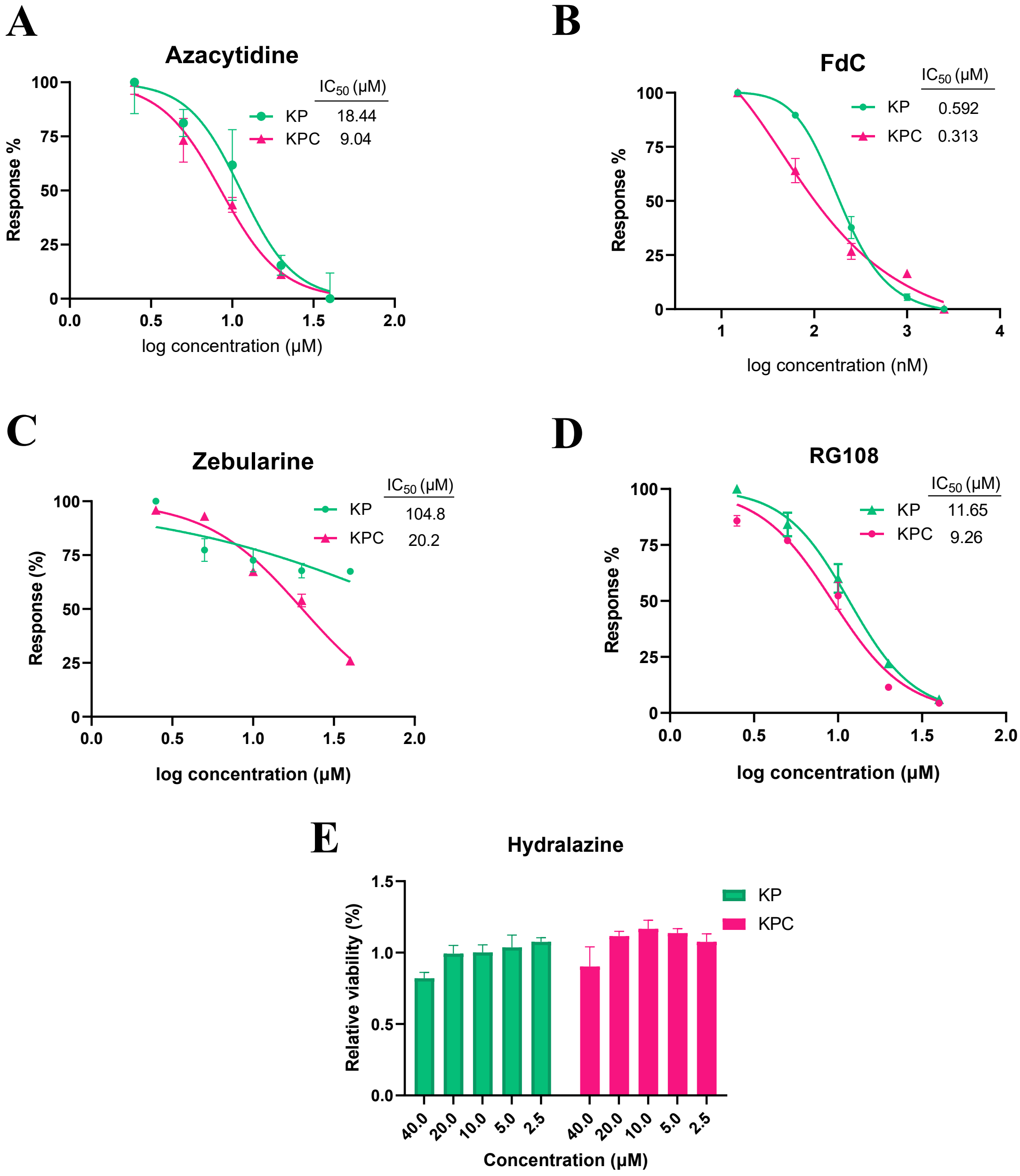

Suppl. Fig. 7. Relative viability results of (A) Azacytidine, (B) FdC, (C) Zebularine, and (D) RG108 (E) Hydralazine on KP and KPC organoids. IC_{50} is the half-maximal inhibitory concentration of the drug in each model. The dose response study was conducted with 750 cells per well using a range of drug concentrations incubated for 5 days to assess cell viability by Cell Titer Glo. Data represents the mean ± standard deviation, n = 3 technical replicates.

A

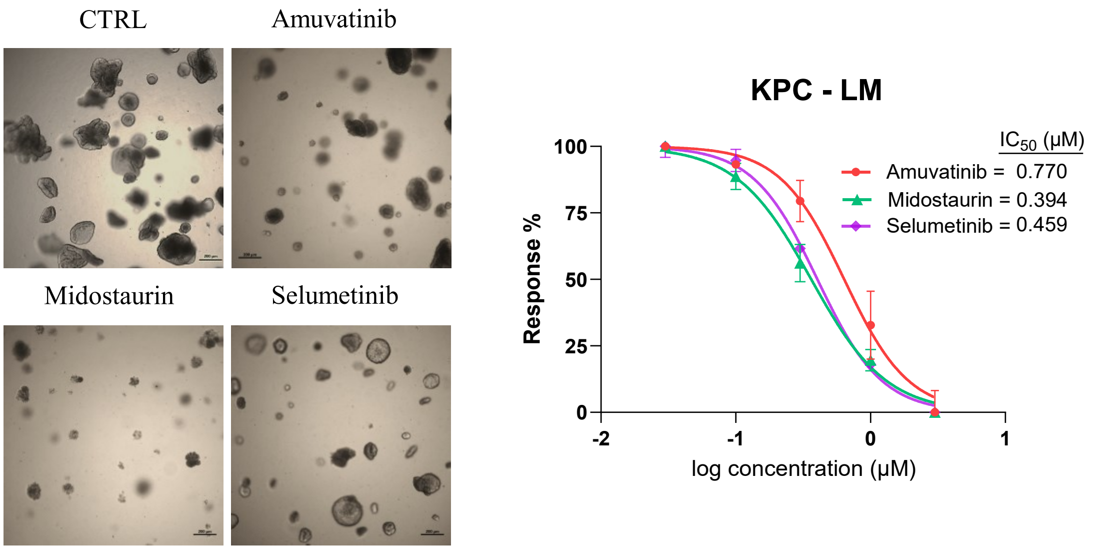

B

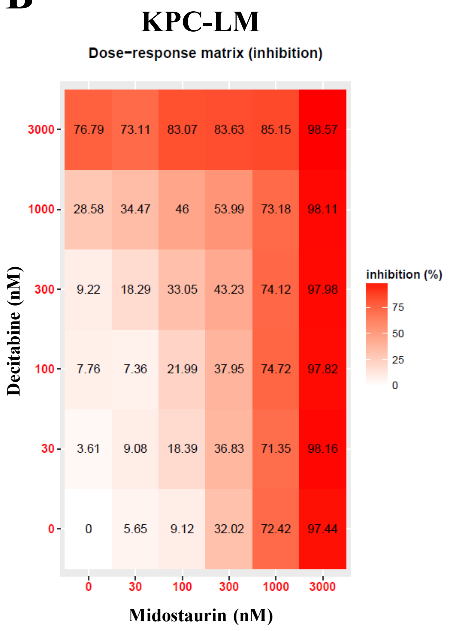

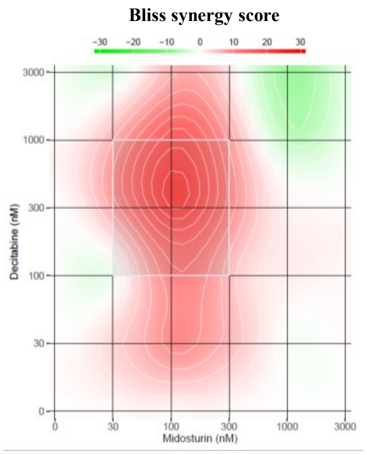

Combination (nM)		Bliss synergy score
Deci	Mido	
30	100	9,7
100	100	10,3
300	100	20,4

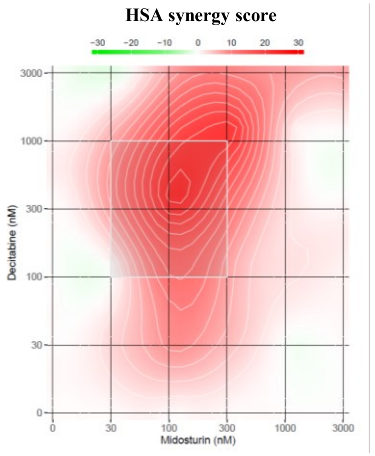

Combination (nM)		HSA synergy score
Deci	Mido	
30	100	9,3
100	100	12,9
300	100	23,8

Suppl. Fig. 8. (A) Representative images and dose-response curves for amuvatinib, midostaurin, and selumetinib on KPC-LM organoids. (B) Combination treatment and synergy analysis of midostaurin with decitabine on KPC-LM organoids. The heatmaps display growth inhibition (%) across different concentration combinations. The highest synergy scores are visualized in synergy maps, highlighting the most effective concentration pairs for synergistic inhibition. The tables below the synergy maps indicate the most synergistic area scores calculated using the Bliss and HSA synergy models. A synergy score between -10 and 10 indicates an additive effect, while scores above 10 suggest synergy. Deci: Decitabine, Mido: Midostaurin

Figure 7.

A

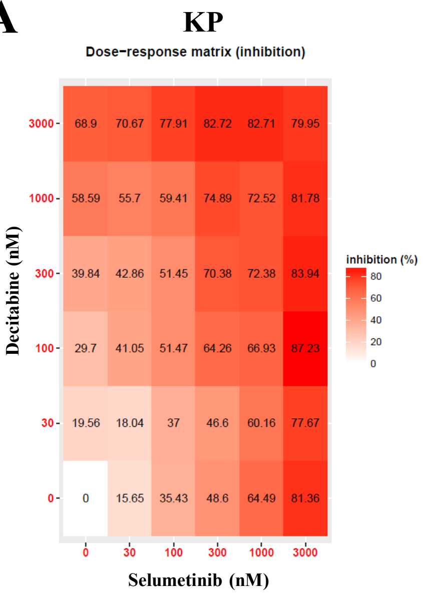

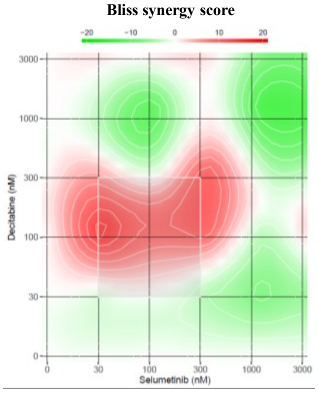

Combination (nM)		Bliss synergy score
Deci	Selu	
100	100	7,6
100	300	9,5
300	300	9,4

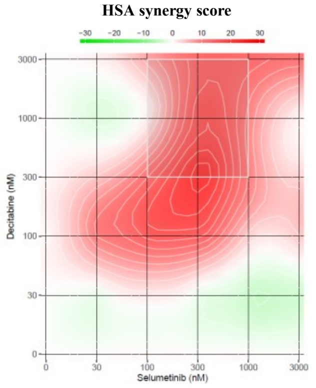

Combination (nM)		HSA synergy score
Deci	Selu	
100	100	16,0
100	300	15,7
300	300	21,8

B

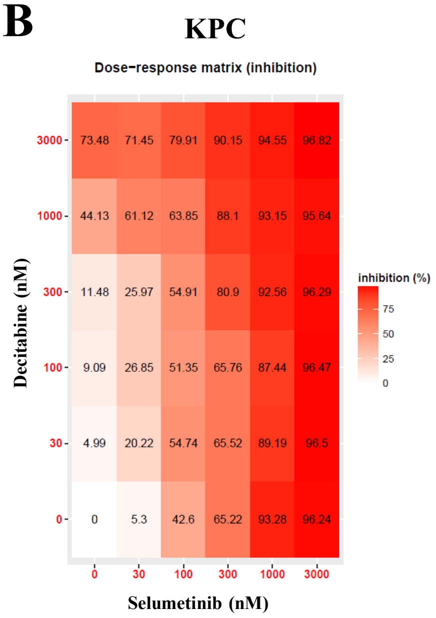

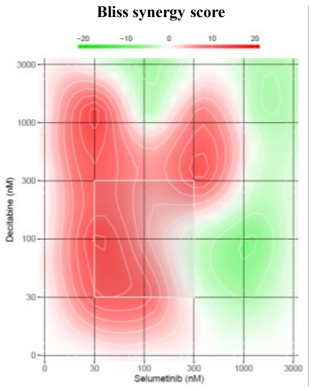

Combination (nM)		Bliss synergy score
Deci	Selu	
30	30	10,2
100	30	12,9
300	30	9,8

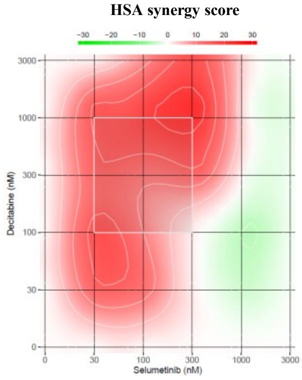

Combination (nM)		HSA synergy score
Deci	Selu	
30	30	14,9
100	30	17,8
300	30	14,5

C

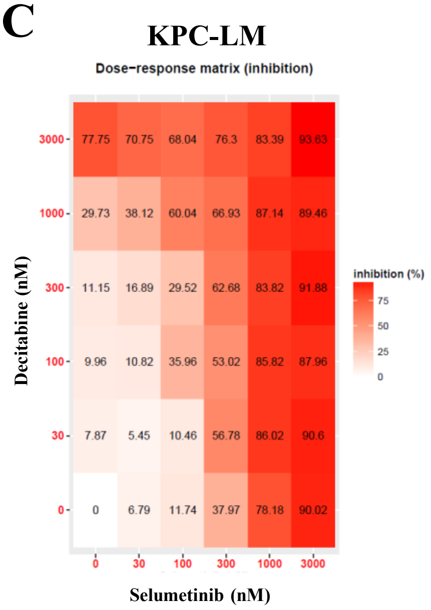

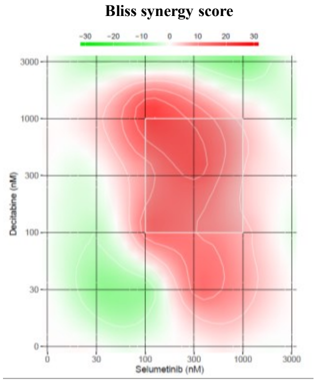

Combination (nM)		Bliss synergy score
Deci	Selu	
100	100	15,4
300	100	7,9
1000	100	22,1

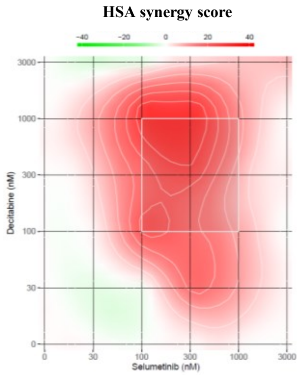

Combination (nM)		HSA synergy score
Deci	Selu	
100	100	24,2
300	100	17,7
1000	100	30,3

Suppl. Fig. 9. Combination treatment and synergy analysis of Selumetinib with Decitabine on (A) KP, (B) KPC, and (C) KPC-LM organoids. The heatmaps display growth inhibition (%) across different concentration combinations. The highest synergy scores are visualized in synergy maps, highlighting the most effective concentration pairs for synergistic inhibition. The tables below the synergy maps indicate the most synergistic area scores calculated using the Bliss and HSA synergy models. A synergy score between -10 and 10 indicates an additive effect, while scores above 10 suggest synergy. Deci: Decitabine, Selu: Selumetinib

A KP

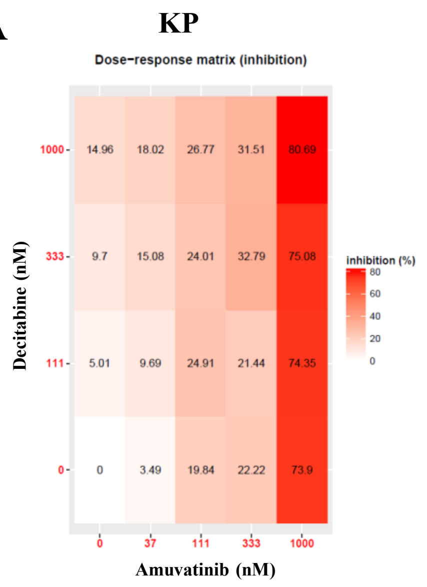

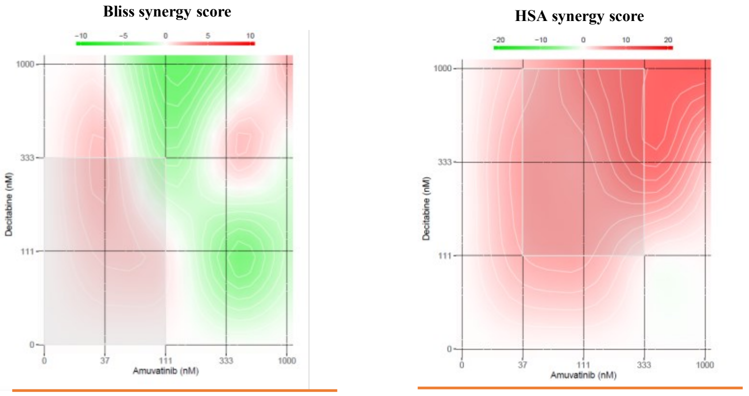

Combination (nM)		Bliss synergy score
Deci	Amu	
111	111	1,1

Combination (nM)		HSA synergy score
Deci	Amu	
111	111	5,1
333	111	4,2
1000	111	6,9

B KPC

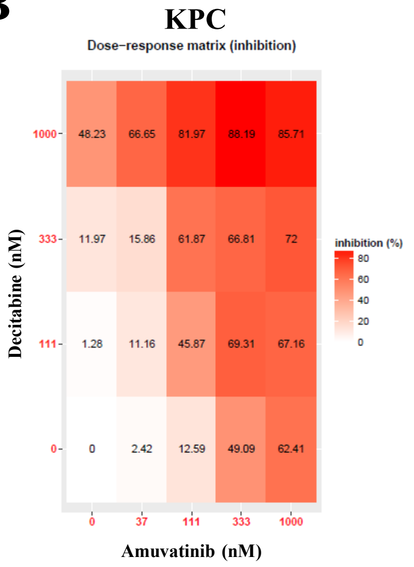

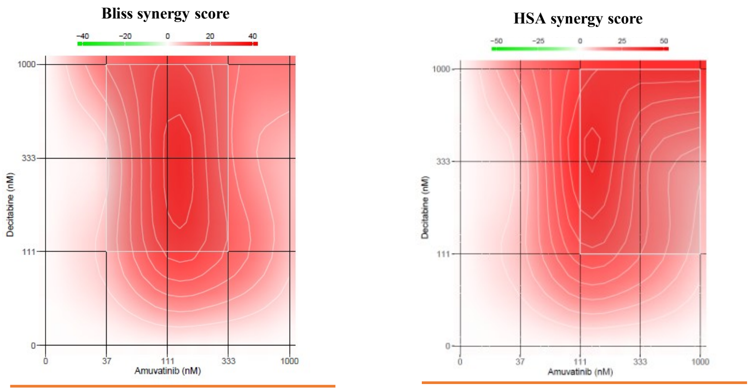

Combination (nM)		Bliss synergy score
Deci	Amu	
111	111	32,2
333	111	38,8
1000	111	27,2

Combination (nM)		HSA synergy score
Deci	Amu	
111	111	33,3
333	111	49,3
1000	111	33,7

C KPC-LM

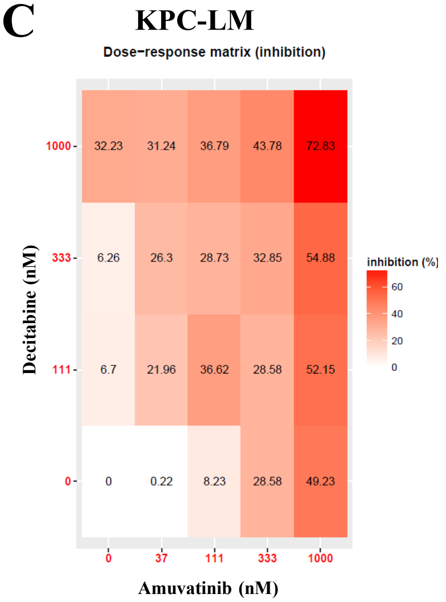

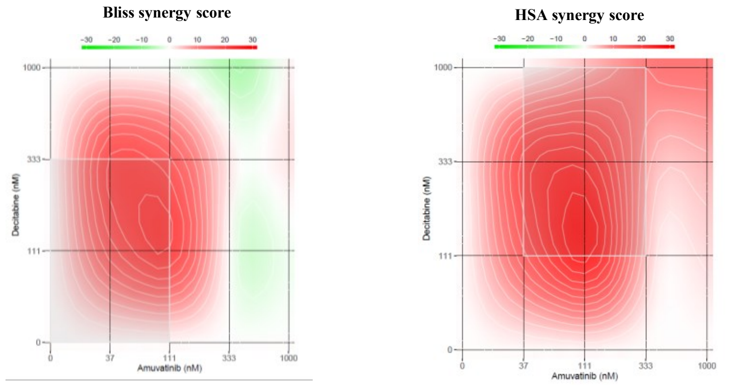

Combination (nM)		Bliss synergy score
Deci	Amu	
111	111	22,2
333	111	14,7

Combination (nM)		HSA synergy score
Deci	Amu	
111	111	28,4
333	111	20,5

Suppl. Fig. 10. Combination treatment and synergy analysis of Amuvatinib with Decitabine on (A) KP, (B) KPC, and (C) KPC-LM organoids. The heatmaps display growth inhibition (%) across different concentration combinations. The highest synergy scores are visualized in synergy maps, highlighting the most effective concentration pairs for synergistic inhibition. The tables below the synergy maps present the most synergistic area scores calculated using the Bliss and HSA synergy models. A synergy score between -10 and 10 indicates an additive effect, while scores above 10 suggest synergy. Deci: Decitabine, Amu: Amuvatinib.

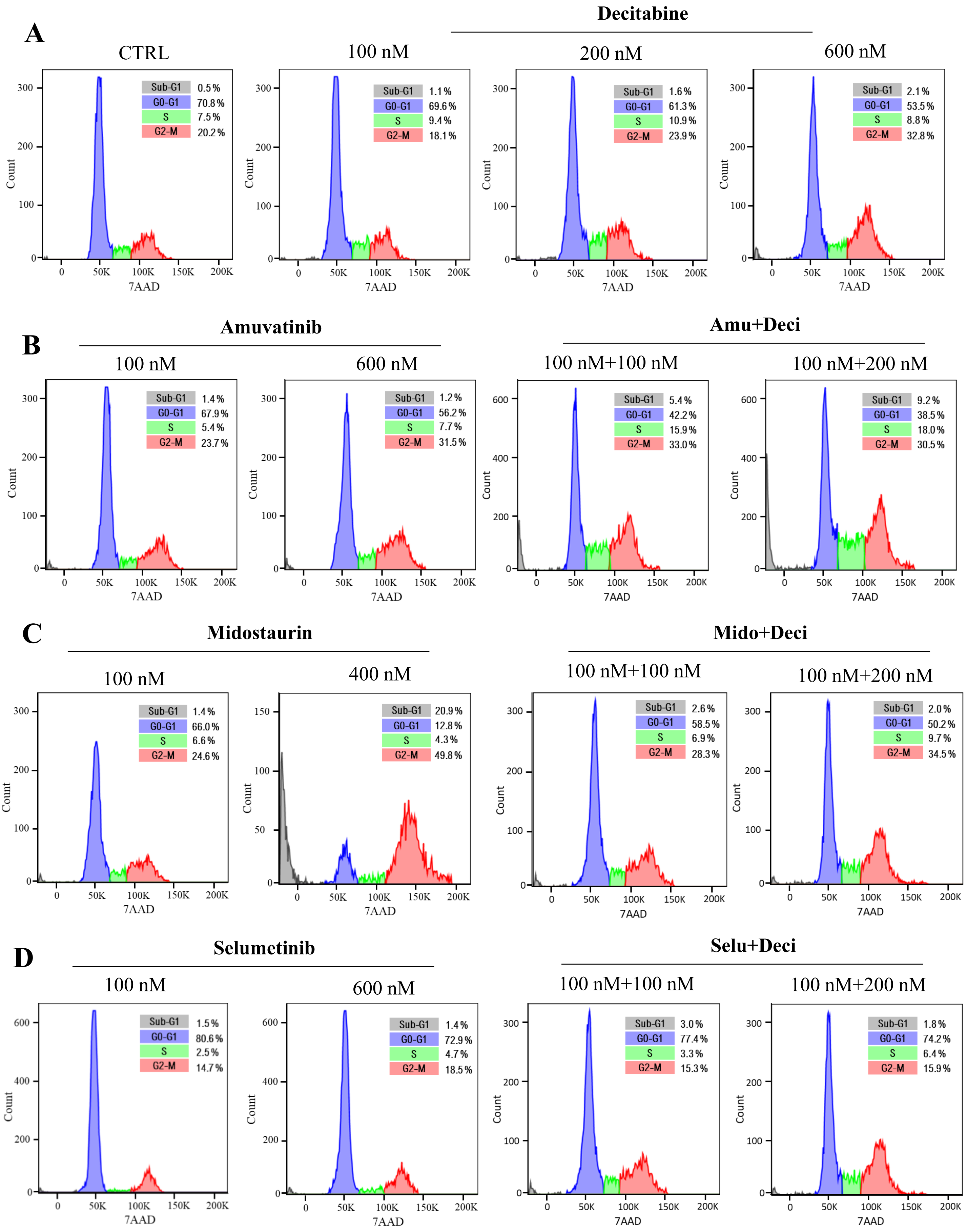

Suppl. Fig. 11. Analysis of cell cycle arrest on KP organoids treated with (A) decitabine, (B) Amuvatinib, (C) midostaurin and (D) selumetinib. Each representative includes the value of Sub-G1, G0-G1, S and G2-M phase of the cell cycle based on the treatments.

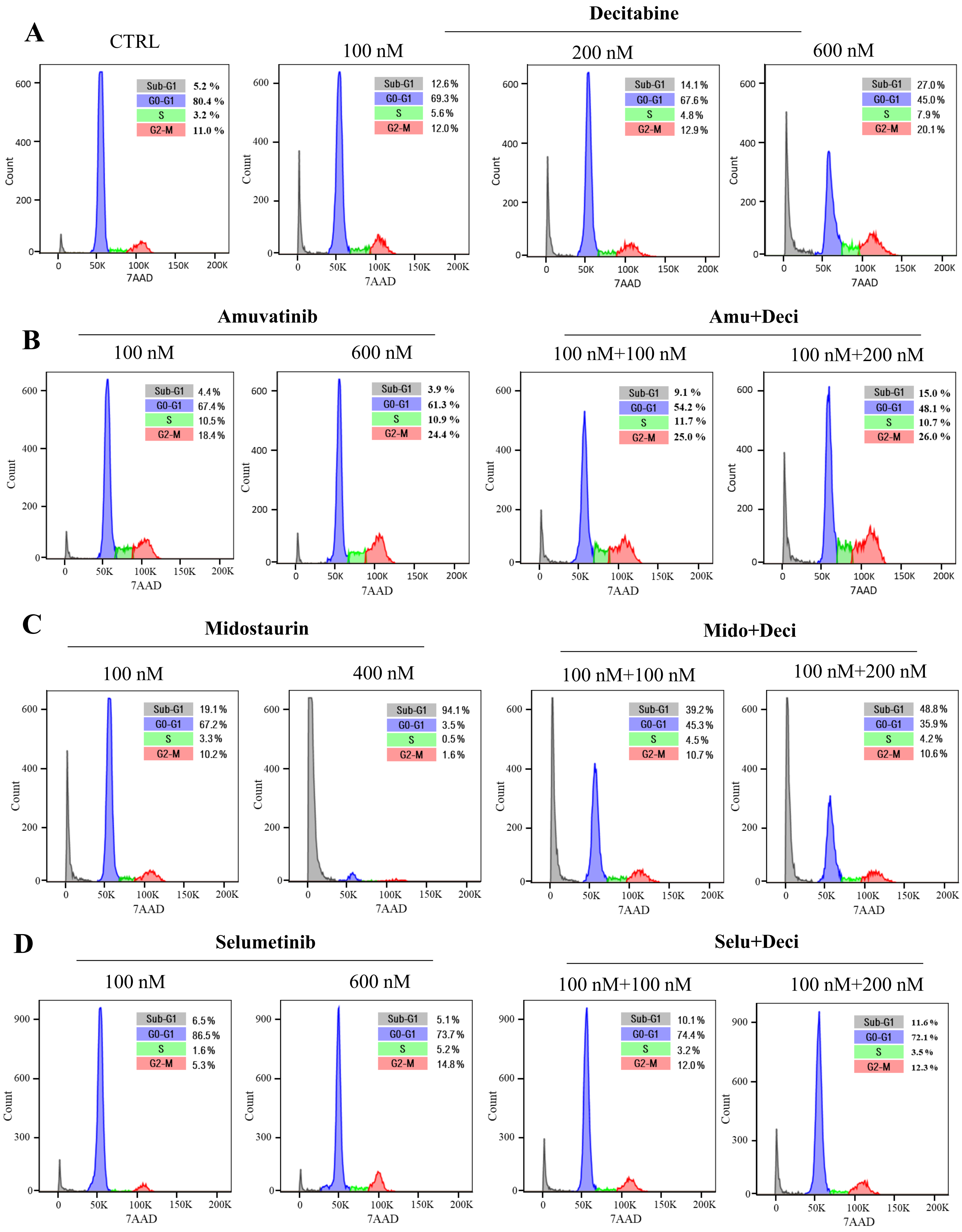

Suppl. Fig. 12. Analysis of cell cycle arrest on KPC organoids treated with (A) decitabine, (B) Amuvatinib, (C) midostaurin and (D) selumetinib. Each representative includes the value of Sub-G1, G0-G1, S and G2-M phase of the cell cycle based on the treatments.

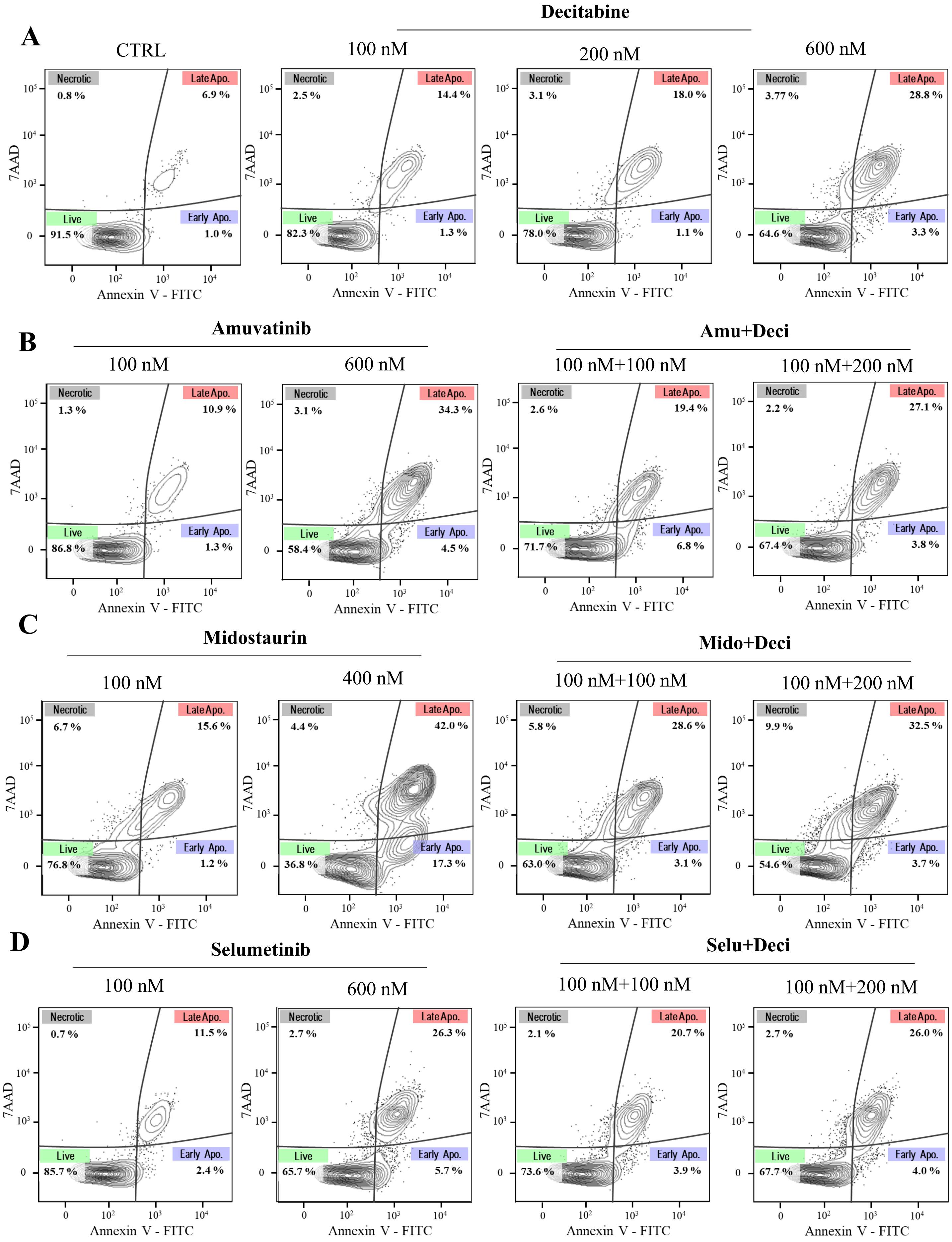

Suppl. Fig. 13. Apoptosis detection by 7AAD and Annexin V on KP organoids treated with (A) decitabine, (B) Amuvatinib, (C) midostaurin and (D) selumetinib. Each representative includes the value of live, necrotic, early and late apoptotic cell populations based on the treatments.

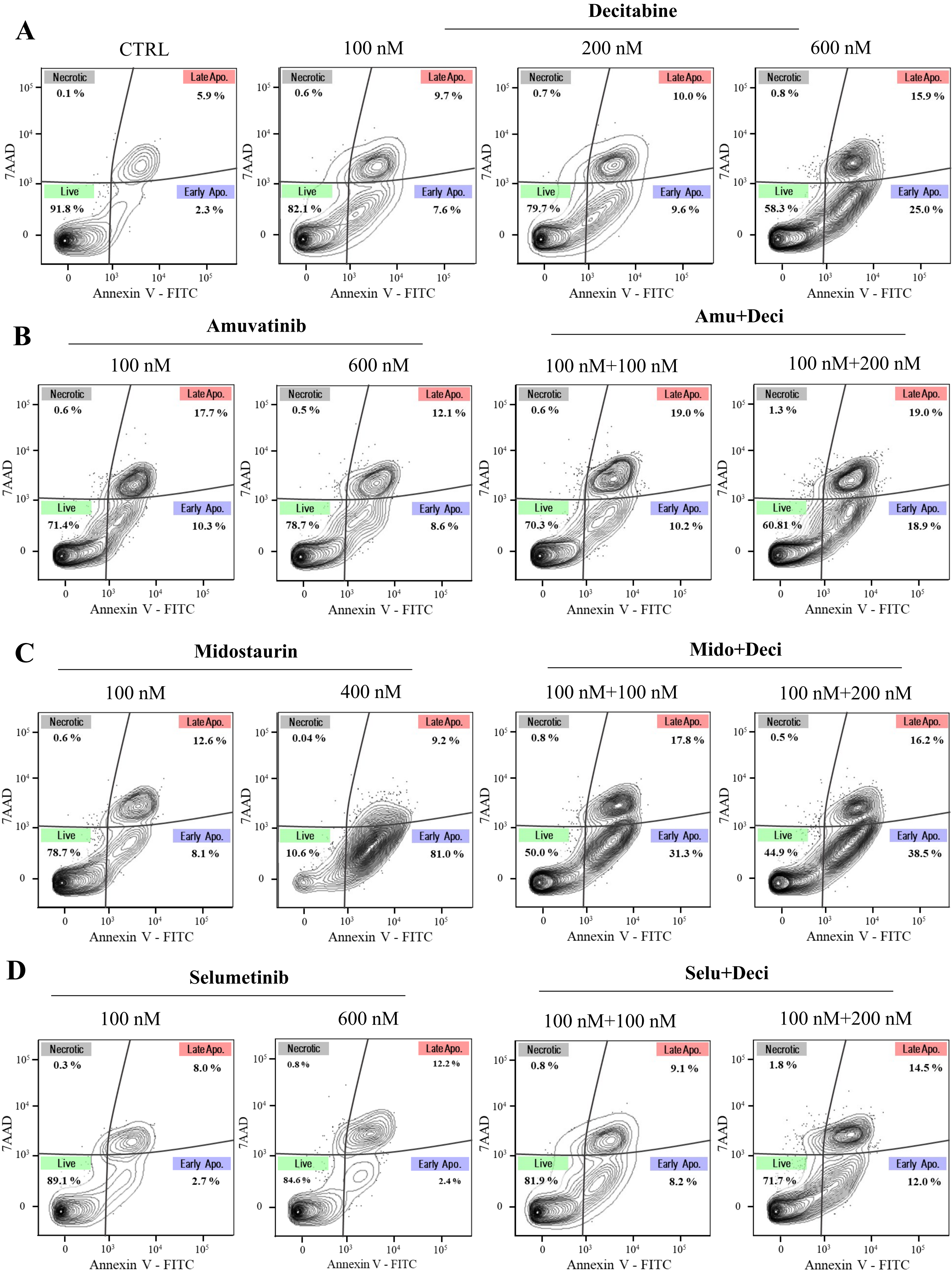

Suppl. Fig. 13. Apoptosis detection by 7AAD and Annexin V on KPC organoids treated with (A) decitabine, (B) Amuvatinib, (C) midostaurin and (D) selumetinib. Each representative includes the value of live, necrotic, early and late apoptotic cell populations based on the treatments.

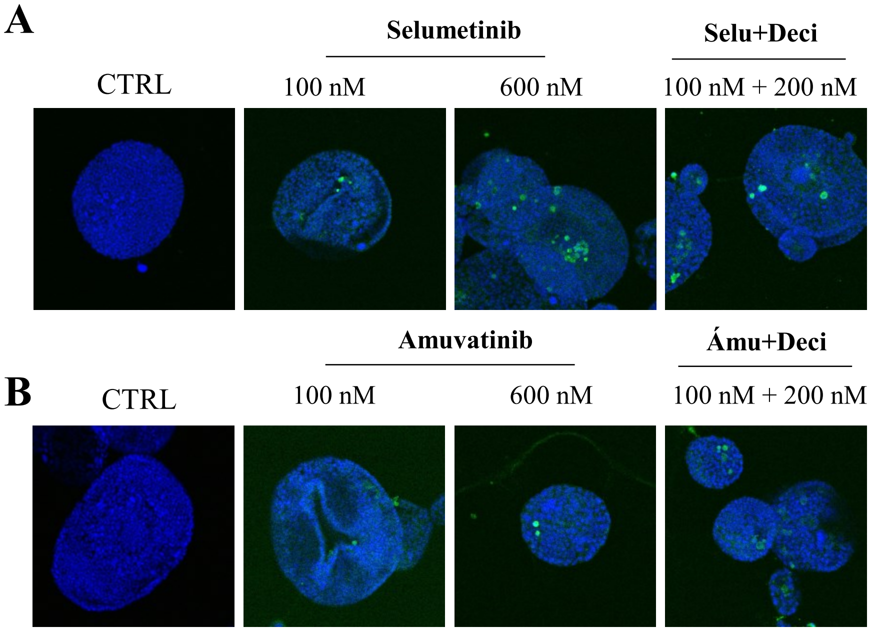

Suppl. Fig. 15: (A) Cleaved-caspase 3 expression in KPC órganoids treated with selumetinib (A) and amuvatinib (B) together with decitabine combination.

Supplementary tables

Suppl Table 1. Lung organoid media composition. Components marked with * are specific to each organoid model; Nutlin-3a is absent in healthy organoids, R-spondin-1 is absent in the KPC model, and fibroblast growth factor 7 and 10 is added to healthy organoid medium.

Reagent name	***Supplier***	***Catalog number***	***Concentration in media***
Advanced DMEM/F12	Thermo fisher Scientific	12634-010	1x
HEPES	Corning	25-060	10 mM
GlutaMAX^{TM} supplement	Thermo Fisher Scientific	35050-061	1x
Antibiotic-Antimycotic (100x)	Thermo Fisher Scientific	15240062	1x
B-27 supplement (50X)	Thermo Fisher Scientific	17504-044	1x
Nicotinamide	Sigma-Aldrich	N0636	10 mM
N-acetyl-L-Cysteine	Biogems	6169116-10G	1.25 mM
Noggin	Peprotech	120-10C	100 ng/ml
A83-01	Biogems	9094360	500 nM
SB202190	Biogems	1523072	10 mM
Nutlin-3a*	Selleck Chemicals	S8059	5 µM
R-Spondin-1*	Peprotech	120-38	500 ng/ml
Fibroblast growth factor 7*	Peprotech	100-19	25 ng/ml
Fibroblast growth factor 10*	Peprotech	100-26	20 ng/ml
Y-27632	Biogems	1293823	10 mM

Suppl. Table 2. Genotyping and sanger sequencing results of organoid lines and their parental tissues.

Tumor(#) & Organoid lines (MLT)	Mutation status	***Ctnnb1***$^{\Delta ex3}$ **PCR**	***Ctnnb1***$^{\Delta ex3}$ **Sanger**	***Kras*** ^{G12V} **Sanger**	***Trp53*** $^{\Delta ex2-10}$ **PCR**	***Trp53*** $^{\Delta ex2-10}$ **Sanger**
#132	*Kras&Trp53*	No	No	Yes	Yes	Yes
MLT5	*Kras&Trp53*	No	No	Yes	Yes	Yes
#174	*Kras&Trp53*	No	No	Yes	Yes	Yes
MLT18	*Kras&Trp53*	No	No	Yes	Yes	Yes
#348	*Kras&Trp53&Ctnnb1*	Yes	Yes	Yes	Yes	Yes
MLT9	*Kras&Trp53&Ctnnb1*	Yes	Yes	Yes	Yes	Yes
#99	*Kras&Trp53&Ctnnb1*	Yes	Yes	Yes	Yes	Yes
MLT3	*Kras&Trp53&Ctnnb1*	Yes	Yes	Yes	Yes	Yes
#99 (Liver met.)	*Kras&Trp53&Ctnnb1*	Yes	Yes	Yes	Yes	Yes
MLT3L	*Kras&Trp53&Ctnnb1*	Yes	Yes	Yes	Yes	Yes
#4	Healthy	No	No	No	No	NA

Suppl. Table 3. Overview of the antibodies used for immunofluorescence.

Primary antibodies	***Dilution***	***Identifier***	***Source***
Rabbit IgG monoclonal anti-TTF1	1:250	ab76013	Abcam
Rabbit IgG monoclonal anti-Cytokeratin 7	1:150	ab181598	Abcam
Rabbit monoclonal [SP27] anti-Cytokeratin 5	1:400	ab64081	Abcam
Rabbit monoclonal [SP6] anti-Ki67	1:250	ab16667	Abcam
Rabbit Polyclonal PD-L1/CD274	1:50	17952-1	Proteintech
Rabbit monoclonal PD-L1 (D5V3B)	1:100	64988	Cell Signaling
Rabbit Polyclonal Cleaved Caspase-3 (Asp175)	1:400	9661	Cell Signaling
Secondary antibody	***Dilution***	***Identifier***	***Source***
Goat anti-Rabbit IgG Alexa Fluor 488	1:1000	A11034	Invitrogen
Goat anti-Rabbit IgG Alexa Fluor 647	1:1000	A-21245	Invitrogen

Suppl. Table 4. List of the targeted drugs and their screenings on *Kras/Trp53* mutated and healthy lung organoids

Pathway	Target	Drug (1 μM)	Inhibition Score: KP organoids	Inhibition Score: Healthy organoids
Ctrl	KRAS	6H05	2	2
Tyrosine Kinase	EGFR	Dacomitinib	2	2
		AG-1478	1	1
		Butein	2	1
		Icotinib	1	1
	HER2	AG-879	1	1
	EGFR/HER2	Lapatinib	1	2
		Afatinib	1	2
	c-Met/ALK	Crizotinib	2	2
	IGF1R/ALK	AZD 3463	3	3
	C-Kit/Bcr-Abl/PDGFR	Imatinib	2	2
	FLT3/c-Kit/c-Met/VEGFR	Cabozantinib	1	1
	FLT3/c-RET/c-Kit/PDGFR	Amuvatinib	2	1
	FLT3/c-Src/c-Kit/ PDGFR/VEGFR/Akt/PKC	Midostaurin	3	2
MAPK	B-RAF V600E	Dabrafenib	1	1
	C-RAF	GW5074	1	1
	Pan-RAF	PLX-4720	1	1
		AZ628	1	2
		Sorafenib	1	2
	MEK	Selumetinib	3	2
		Refametinib	3	3
PI3K	mTOR	AZD8055	3	3
		Palomid	1	1
		WYE-354	1	1
		WYE-132	3	3
	mTOR/PI3K	Dactolisib	3	3

Suppl. Table 5. List of the epigenetic drugs clasified as DNMT inhibitors and their background.

Status	*Drug*	*Generation*	*Mechanism of action*	*Key differences*	*Ref.*
**FDA approved**	Azacytidine	First	-incorporates into DNA and RNA<br>-leading to hypomethylation<br>-reactivation of silenced genes	Incorporates into both DNA and RNA.	18
	Decitabine	First	-incorporates into DNA,<br>-leading to hypomethylation<br>-reactivation of silenced genes	Incorporates only into DNA, more potent than Azacytidine.	18
**In clinical research**	5-Fluoro-2’-deoxycytidine (FdCyd)	Second	-Decitabine analog,<br>-incorporated into DNA,<br>-leading DNA hypomethylation<br>-reactivation of silenced genes	A fluorinated analog, potentially more effective and less toxic.	18
	Hydralazine	First	-originally an antihypertensive<br>-leading to hypomethylation<br>-reactivation of silenced genes.	Off-target effects due to its original antihypertensive function.	18
**In preclinical research**	Zebularine	Second	-Azacytidine derivative<br>-incorporates into DNA<br>-leading to hypomethylation<br>-reactivation of silenced genes	Improved stability and reduced toxicity compared to Azacytidine and Decitabine.	18
	RG108	Second	-Non-nucleoside DNMTi<br>-binds to the catalytic site of DNMTs,<br>-preventing DNA methylation without incorporating into DNA	Does not incorporate into DNA, which reduces potential side effects associated with nucleoside analogs.	18
